# Transmission of amyloid-beta and tau pathologies is associated with cognitive impairments in a primate

**DOI:** 10.1186/s40478-021-01266-8

**Published:** 2021-10-12

**Authors:** Suzanne Lam, Fanny Petit, Anne-Sophie Hérard, Susana Boluda, Sabiha Eddarkaoui, Martine Guillermier, The Brain Bank Neuro-C. E. B. Neuropathology Network, Franck Letournel, Marie-Laure Martin-Négrier, Maxime Faisant, Catherine Godfraind, Jean Boutonnat, Claude-Alain Maurage, Vincent Deramecourt, Mathilde Duchesne, David Meyronet, Tanguy Fenouil, André Mauès de Paula, Valérie Rigau, Fanny Vandenbos-Burel, Danielle Seilhean, Charles Duyckaerts, Susana Boluda, Isabelle Plu, Dan Christian Chiforeanu, Annie Laquerrière, Florent Marguet, Béatrice Lannes, Benoît Lhermitte, Luc Buée, Charles Duyckaerts, Stéphane Haïk, Jean-Luc Picq, Marc Dhenain

**Affiliations:** 1 Laboratoire des Maladies Neurodégénératives, Université Paris-Saclay, CEA, CNRS, 18 Route du Panorama, 92265 Fontenay-aux-Roses, France; 2 Commissariat à l’Energie Atomique et aux Energies Alternatives (CEA), Direction de la Recherche Fondamentale (DRF), Institut François Jacob, MIRCen, 18 Route du Panorama, 92265 Fontenay-aux-Roses, France; 3 Institut du Cerveau, UMR 7225, Sorbonne Université, Paris Brain Institute-ICM, CNRS, AP-HP, Hôpital de la Pitié Salpêtrière, Inserm U1127 DMU Neurosciences, Paris, France; 4 Brainbank NeuroCEB Neuropathology Network : Plateforme de Ressources Biologiques, Hôpital de La Pitié-Salpêtrière, Bâtiment Roger Baillet, 47-83 boulevard de l’Hôpital, 75651 Paris Cedex 13, France; 5 Université de Lille, CHU-Lille, LabEx DISTALZ, Alzheimer & Tauopathies, Rue Polonovski, 59045 Lille, France; 6 Laboratory of Cognitive Functioning and Dysfunctioning (DysCo), University of Paris 8, 93526 cedex Saint-Denis, France; 7 Angers, France; 8 Bordeaux, France; 9 Caen, France; 10 Clermont-Ferrand, France; 11 Grenoble, France; 12 Lille, France; 13 Limoges, France; 14 Lyon, France; 15 Marseille, France; 16 Montpellier, France; 17 Nice, France; 18 Paris, France; 19 Rennes, France; 20 Rouen, France; 21 Strasbourg, France

**Keywords:** Amyloid-β pathology, Alzheimer's disease, Cerebral atrophy, Prion, Tau pathology, Transmission

## Abstract

Amyloid-β (Aβ) pathology transmission has been described in patients following iatrogenic exposure to compounds contaminated with Aβ proteins. It can induce cerebral Aβ angiopathy resulting in brain hemorrhages and devastating clinical impacts. Iatrogenic transmission of tau pathology is also suspected but not experimentally proven. In both scenarios, lesions were detected several decades after the putatively triggering medico-surgical act. There is however little information regarding the cognitive repercussions in individuals who do not develop cerebral hemorrhages. In the current study, we inoculated the posterior cingulate cortex and underlying corpus callosum of young adult primates (*Microcebus murinus*) with either Alzheimer’s disease or control brain extracts. This led to widespread Aβ and tau pathologies in all of the Alzheimer-inoculated animals following a 21-month-long incubation period (n = 12) whereas none of the control brain extract-inoculated animals developed such lesions (n = 6). Aβ deposition affected almost all cortical regions. Tau pathology was also detected in Aβ-deposit-free regions distant from the inoculation sites (*e.g.* in the entorhinal cortex), while some regions adjacent, but not connected, to the inoculation sites were spared (*e.g.* the occipital cortex). Alzheimer-inoculated animals developed cognitive deficits and cerebral atrophy compared to controls. These pathologies were induced using two different batches of Alzheimer brain extracts. This is the first experimental demonstration that tau can be transmitted by human brain extracts inoculations in a primate. We also showed for the first time that the transmission of widespread Aβ and tau pathologies can be associated with cognitive decline. Our results thus reinforce the need to organize a systematic monitoring of individuals who underwent procedures associated with a risk of Aβ and tau iatrogenic transmission. They also provide support for Alzheimer brain-inoculated primates as relevant models of Alzheimer pathology.

**Supplementary Information:**

The online version contains supplementary material available at 10.1186/s40478-021-01266-8.

## Introduction

Prion diseases can occur after iatrogenic transmission of misfolded prion proteins. The aberrant proteins propagate by imposing their abnormal conformation on the homologous normal host cell proteins which are continually produced in the natural course of cellular metabolism. Neuropathological observational studies in humans have suggested that amyloid-β (Aβ) pathology is transmissible through a similar mechanism to that of acquired prion diseases [17]. To date, 76 cases of Aβ pathology have been reported following exposure to cadaver-sourced human growth hormones [9, 17], dura mater grafts [14] or after cerebral surgeries with instruments contaminated with Aβ [9, 21]. The pathology occurred as Aβ plaques in the brain as well as vascular Aβ pathology that could be associated with fatal cerebral hemorrhages. Observational evidence of tau iatrogenic transmission is not as widely reported as for Aβ. A recent article detected tau lesions after incubation periods exceeding three decades in patients with iatrogenic Aβ pathology [16]. However, because of the long incubation time in humans, it remains difficult to determine whether the Aβ and tau pathologies were really transmitted. Another critical question is whether, in the absence of severe cerebral hemorrhages, Aβ and/or tau transmissions can lead to cognitive impairments.

Experimental studies in transgenic mice overexpressing Aβ precursor protein (APP) have shown transmissibility of Aβ pathology after the intracerebral inoculation of Alzheimer's disease (AD) brain extracts [26]. Transmission of tau pathology is also described in mice overexpressing mutated tau proteins [6]. The host in which the proteopathic seeds are inoculated provides the biochemical and physiological environment that modulates lesion emergence and functional impact [18]. Transgenic mouse models of Aβ or tau pathology rely on high Aβ production or mutated tau protein expression, respectively. Thus, one key limitation of these models is that they provide a very different brain environment from the one found in human brains. Because of their phylogenetic proximity, primates have a brain environment closer to the human brain. A long-term study on marmosets (*Callithrix jacchus*) revealed the induction of sparse amyloidosis 3.5 years after intracerebral inoculations of AD brain homogenates [2, 23, 34]. However, tau pathologies or other AD-like features were not reported, even seven years later.

Mouse lemurs (*Microcebus murinus*) are small primates with an Aβ_1-42_ sequence that is homologous to that of humans [37], while mice differ by three amino acids [8]. Protein sequence issued from gene for Microtubule Associated Protein Tau (MAPT) has 94.3% identity with human gene, while murine MAPT gene has 88.8% identity (Additional File 1: Fig. S1a). Recently, our group showed that following an 18-month-incubation period, AD brain inoculations in the hippocampus and overlying cortex of mouse lemurs can lead to cognitive decline, functional alterations and cerebral atrophy associated with neuronal loss, but very sparse Aβ and tau deposits [11]. In the present study, we inoculated the posterior cingulate cortex and underlying corpus callosum of young adult mouse lemurs with either AD or control brain extracts. The posterior cingulate cortex was chosen as it is a functional cerebral hub in mouse lemurs [10]. The corpus callosum was chosen based on the assumption that migration of seeds could follow white matter tracts. As fragmentation of Aβ seeds by extended sonication was shown to increase seeding capacity of brain extracts [20], we carefully sonicated the inoculated samples. Following a 21-month incubation period, all of AD-inoculated mouse lemurs (n = 12) developed extensive Aβ and tau pathologies in several brain regions, providing evidence for the spreading of these pathologies in the brain. Animals inoculated with control-brain extracts did not develop any Aβ plaques or tau deposits. Similar results were replicated using two different types of AD-brain extracts. AD-inoculated animals also developed progressive cognitive impairments and cerebral atrophy.

## Results

### Tau isoforms in mouse lemurs

In humans, MAPT gene can produce a variety of isoforms by alternative splicing. In normal adult human brain there are six isoforms that differ by sequences from exons 2 and 3 that encode N-terminal sequences, and exon 10 that encodes a microtubule binding repeat sequence. When this latter exon is present there are four microtubule binding repeats (4R-tau) and when absent there are three microtubule-binding repeats (3R-tau) [24]. In adult humans, all six brain isoforms are present leading to the presence of both 4R and 3R tau [15]. In adult wild-type mice, the 4R tau is the only isoform [24]. Analysis of tau isoforms in mouse lemurs by immunoblots showed that mouse lemurs present with both the 4R and 3R isoforms, while the isoforms corresponding to exon 2 were not detected due to either their absence or differences in the protein sequence (Additional File 1: Fig. S1b).

### Characterization and inoculation of human brain homogenates

We prepared two brain homogenates from sporadic AD patients, with each homogenate consisting of a combination of four brain extracts from patients with either a slowly evolving form of AD (defined by a disease duration of 5 to 8 years (AD1)) or a rapidly evolving form of AD (defined by a disease duration of 6 months to 3 years (AD2)). A third "control" homogenate, was prepared from the brains of two non-demented individuals (Ctrl). The characteristics of the selected subjects are presented in Additional File 1: Table S1 and Additional File 1: Fig. S2. The amount of Aβ, tau and neuroinflammatory proteins differed slightly between the brain homogenates, as the AD2 brain extract displayed more total tau and phospho-tau181, but less Aβ_38_ and Aβ_40_ than the AD1 one (Additional File 1: Fig. S2g-l). Iba1 and GFAP levels were similar in the two AD homogenates (Additional File 1: Fig. S2m-o). Brain homogenates were bilaterally inoculated into the posterior cingulate cortex and underlying corpus callosum of young-adult 1.5-year-old mouse lemurs (6.25 µl/site, n = 6/group). This corresponded to 33.75, 34.56 and 34.37 µg of total proteins per site, respectively for the Ctrl, AD1 and AD2 groups. This corresponds to 0, 0.75 and 1 pg of Aβ42 and 0, 0.063 and 0.125 µg of phospho-tau181. As depicted in Additional File 1: Fig. S3 showing regions connected to the posterior cingulate, the needle tract passed through the medial part of the parietal cortex (Broadman area 7) [27].

### Aβ pathology induction and spreading after AD brain inoculation

**Fig. 1:**
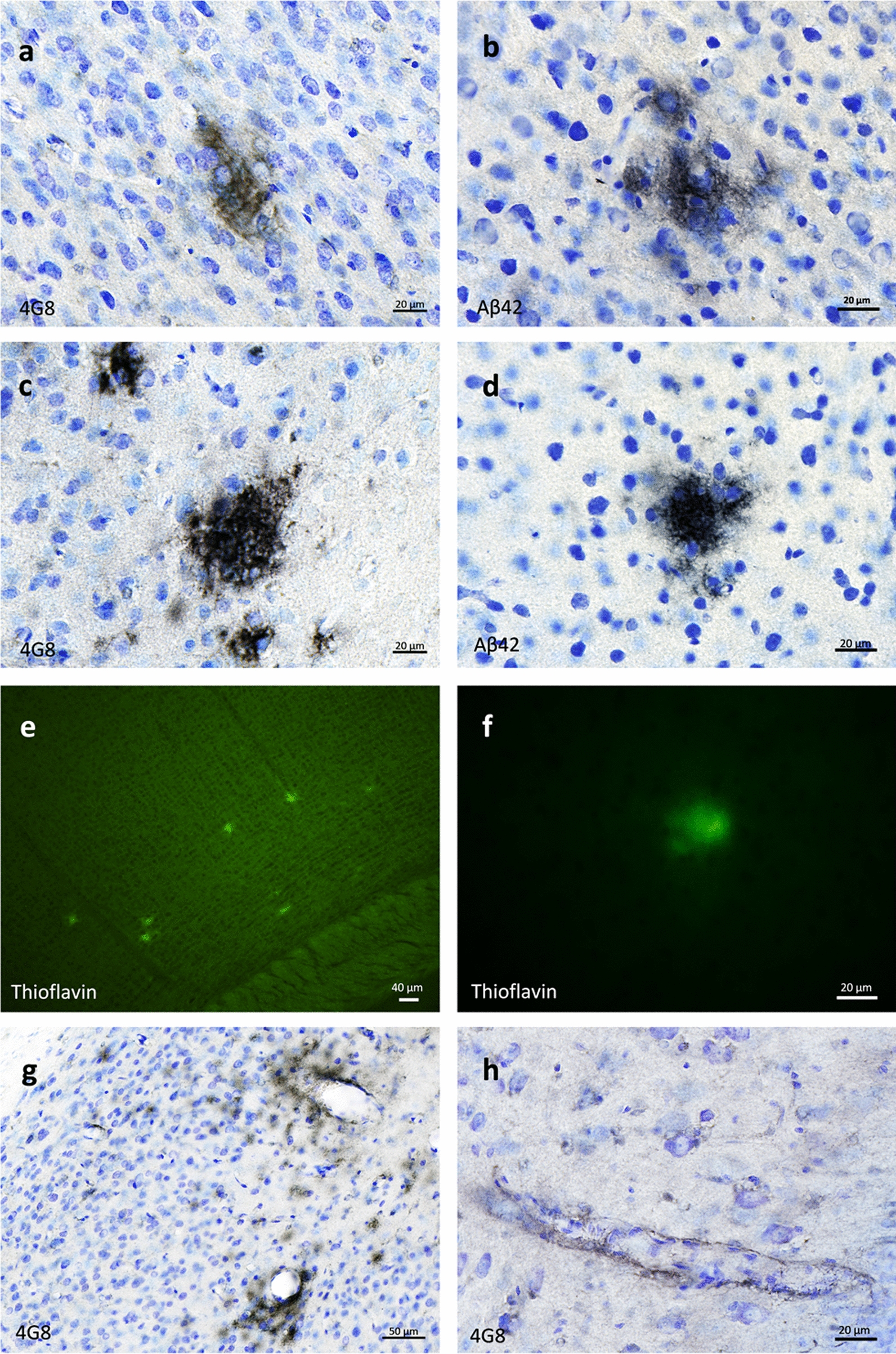
Aβ plaques and vascular deposits in AD-inoculated mouse lemurs. Parenchymal diffuse **a**-**b** and dense **c**-**d** plaques stained by 4G8 or Aβ42 antibodies in AD-brain inoculated mouse lemurs. Plaques were also Thioflavin S-positive **e**–**f**. Cerebral Aβ angiopathy was also observed in cortical regions **g** and in the hippocampus **h** (4G8 antibody). Scale bars: 20 µm in a-d, f, h; 40 µm in e; 50 µm in g

**Fig. 2:**
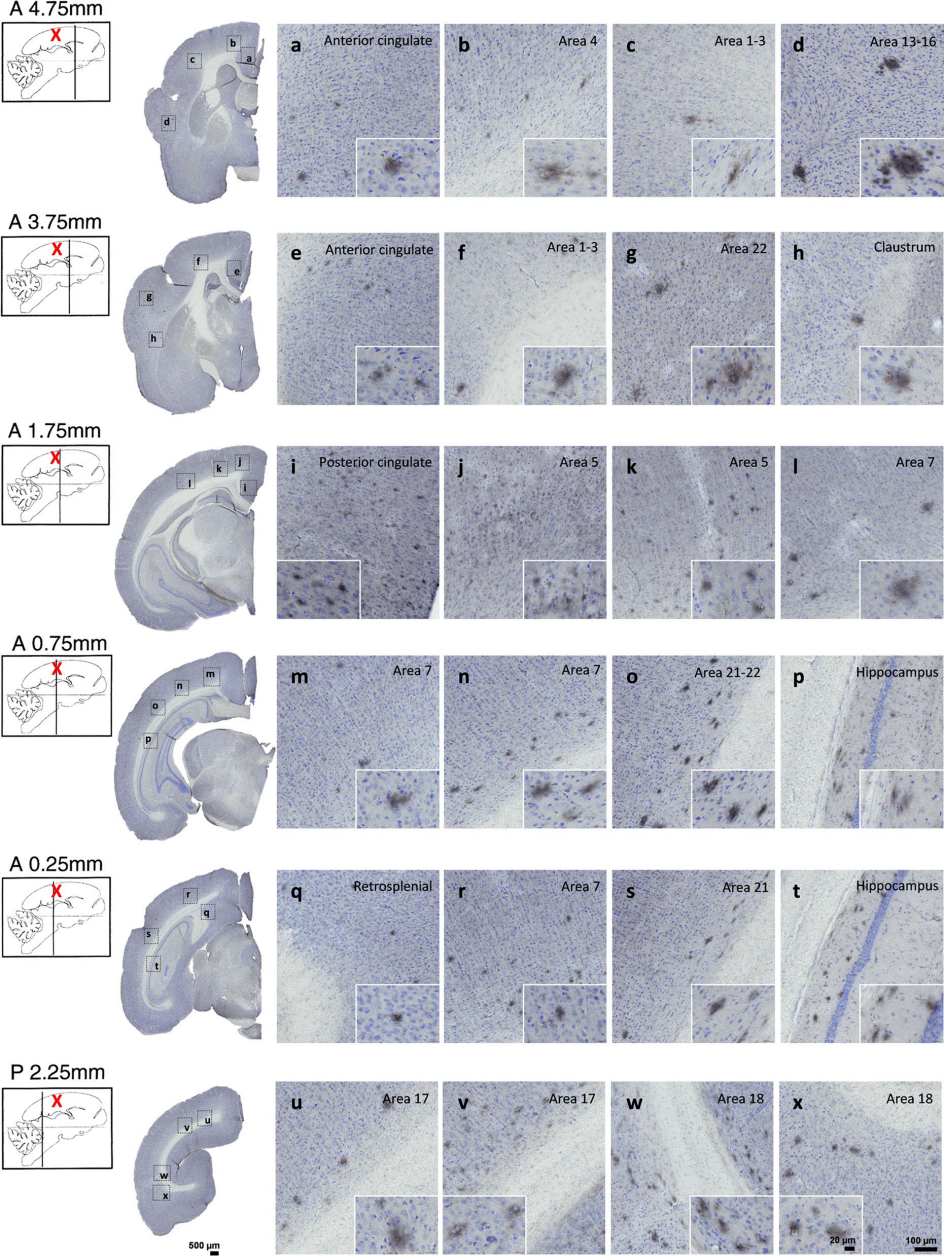
Aβ pathology throughout the brains of mouse lemurs inoculated with AD brain extracts. Representative images of 4G8 immunolabelling showing Aβ pathology throughout the brains of mouse lemurs following AD brain extracts inoculations. Aβ deposition was observed in the inoculated posterior cingulate cortex (i) and around the needle tract in the parietal cortex (area 7; **l**-**n**, **r**). Adjacent regions including the retrosplenial cortex (**q**), parietal area 5 (**j**-**k**) and anterior cingulate cortex (**a**, **e**) also displayed Aβ pathology. Additionally, Aβ plaques were detected in the hippocampus (**p**, **t**) and other regions more distant from the inoculation sites including the superior temporal cortex (areas 22 and 21; **g**, **o**, **s**), the parietal areas 1–3 (**c**, **f**) and the frontal cortex (area 4; **b**). Occipital areas also displayed Aβ deposits (areas 17 and 18; **u**-**x**). The red crosses indicate the inoculated region. Scale bars: 500 µm in whole slice images, 100 µm in zooms and 20 µm in inserts

**Fig. 3:**
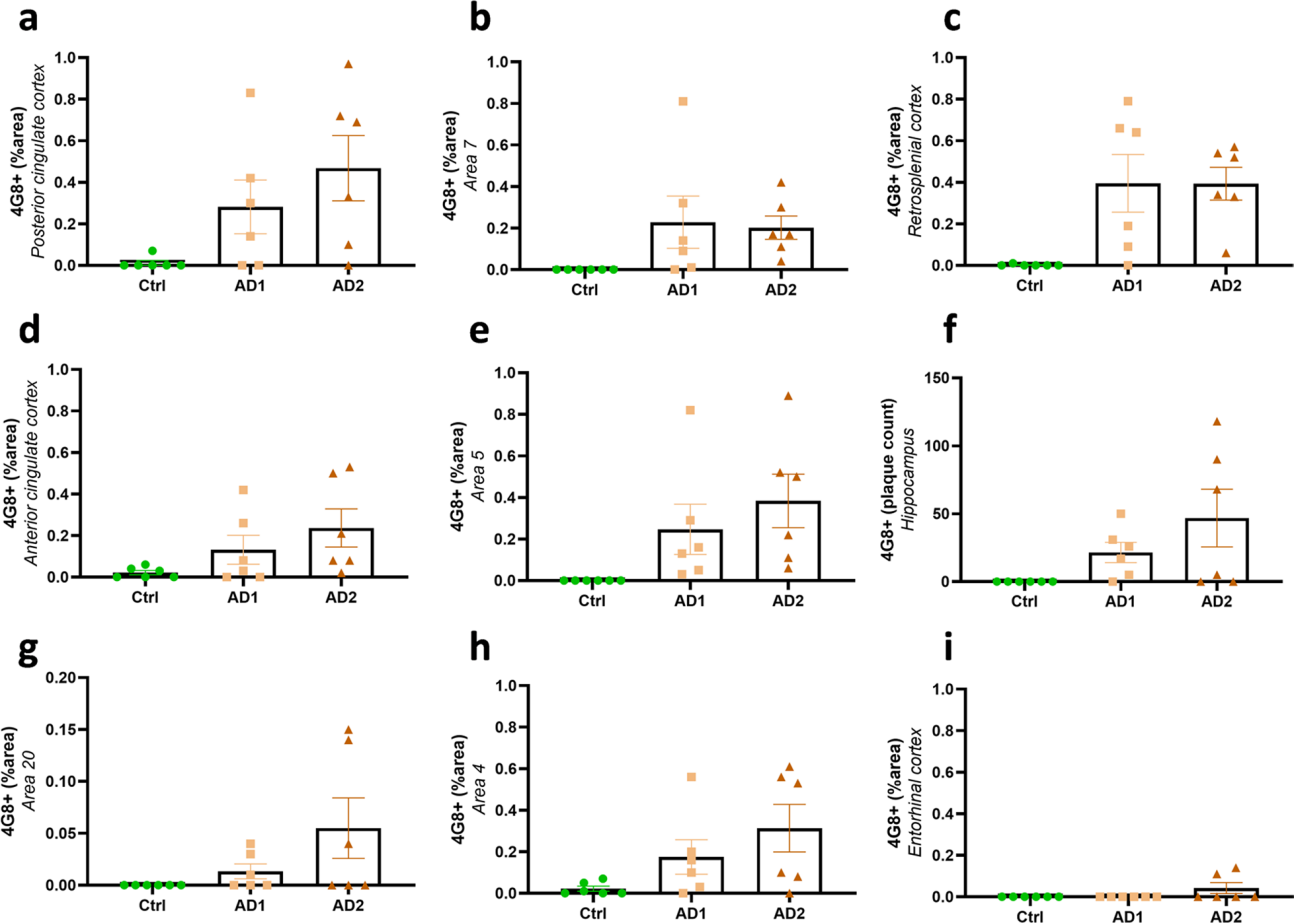
Quantification of Aβ deposition following human brain inoculation*.* Quantification of Aβ load (% 4G8-positive area or Aβ plaque count for the hippocampus) in AD- and Ctrl-inoculated lemurs. Aβ was detected in all of AD-inoculated animals and in none of Ctrl-inoculated lemurs. Some AD-inoculated animals did not display significant amounts of Aβ in some brain regions (*e.g.* two AD1-inoculated animals did not develop Aβ deposits in the posterior cingulate cortex **a**), but displayed more Aβ in other regions. Thus, all animals displayed Aβ deposits in at least some regions of their brains. Altogether, animals from both AD-inoculated groups showed Aβ deposition at the inoculation site (posterior cingulate cortex; **a**) and/or around the needle tract in the parietal cortex (area 7; **b**). Adjacent regions such as the retrosplenial cortex (**c**), anterior cingulate cortex (**d**) and parietal area 5 (**e**) also displayed Aβ pathology. Aβ plaques were also detected in the hippocampus (**f**) and other regions more distant from the inoculation sites including the superior temporal cortex (area 20; **g**) and the frontal cortex (area 4; **h**). The entorhinal cortex (**i**) was the only cortical region that did not display Aβ pathology in any groups. No statistical difference was observed between AD1 and AD2-inoculated animals (*p* > 0.05; Mann–Whitney’s test). Data are shown as mean ± s.e.m

**Fig. 4:**
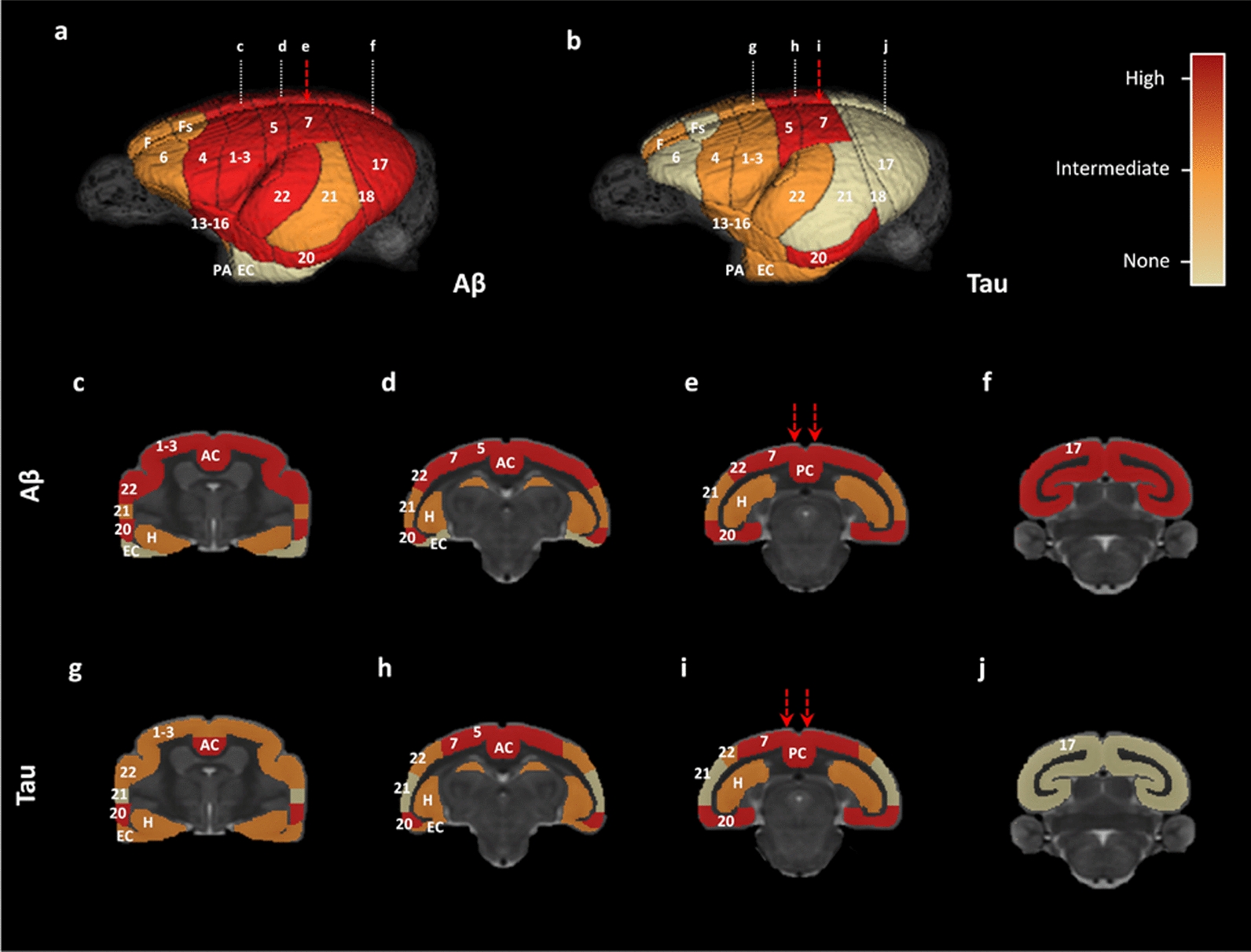
Overview of Aβ and tau pathology spreading throughout the brains of mouse lemurs inoculated with AD brain extracts*.* Three-dimensional rendering of Aβ and tau pathologies in mouse lemur brains using a three-level semi-quantitative scale (no lesions/intermediate/high lesion load). (**a**, **c**-**f**) Aβ pathology involved almost all cortical regions, except for the entorhinal cortex. The hippocampus was also Aβ-positive. (**b**, **g**-**j**) High levels of tau lesions were reported at the inoculated sites (posterior cingulate cortex, area 7) as well as in juxtaposing regions such as the area 5. Distant regions such as the area 20 also displayed high tau pathology. The hippocampus, entorhinal cortex, and other cortical areas displayed intermediate tau lesion loads. The red arrows indicate the needle tracts. Numbers represent Brodmann areas as reported in the mouse lemur brain by (Le Gros Clark, 1931). AC: anterior cingulate cortex, EC: entorhinal cortex, F: antero-medial frontal cortex, Fs: superior frontal cortex, H: hippocampus, PA: peri-amygdalar cortex, PC: posterior cingulate cortex

**Fig. 5:**
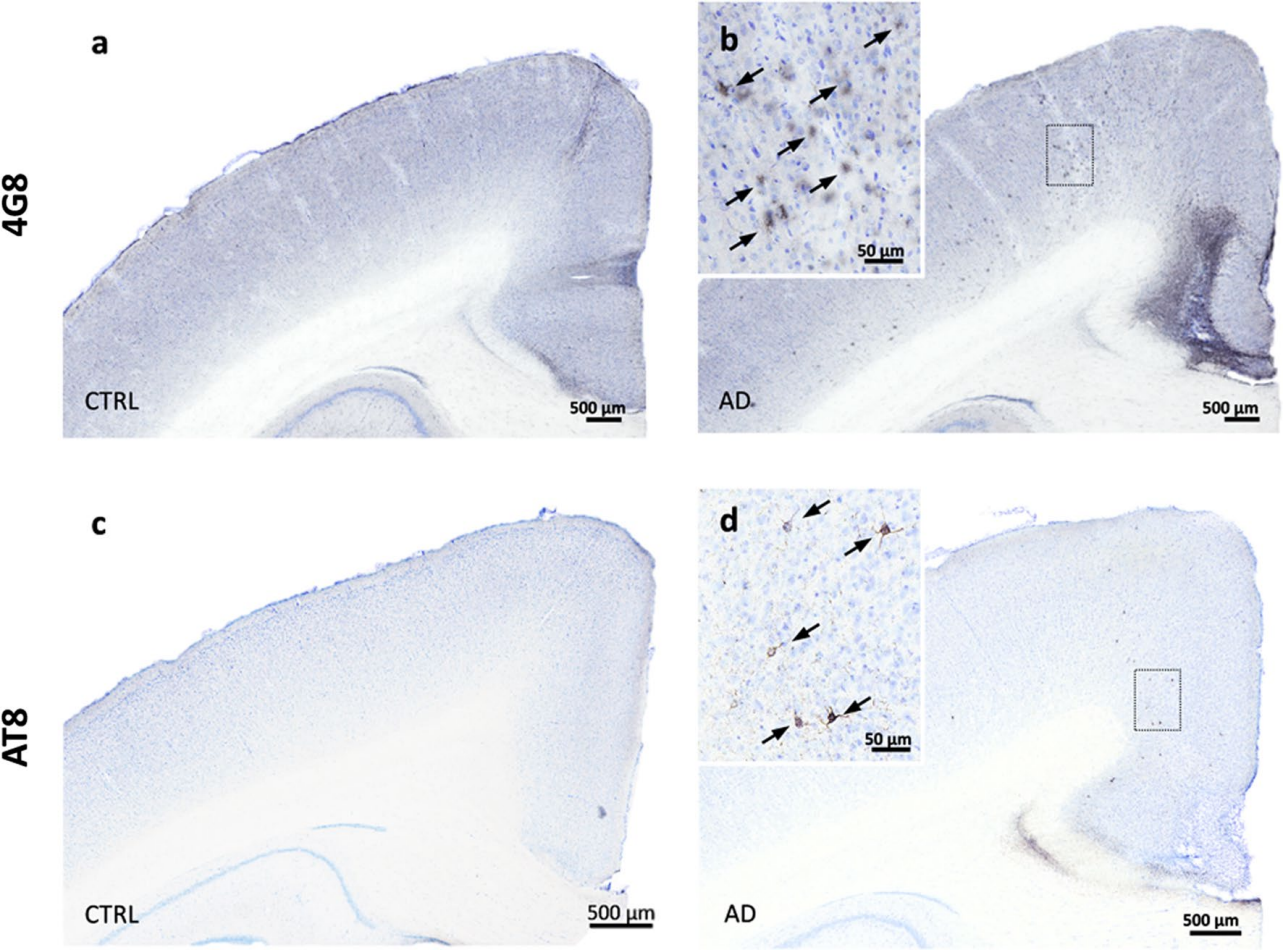
Aβ and tau pathologies were not induced following Ctrl-brain extract inoculations. Representative sections of 4G8 (**a**-**b**) and AT8 (**c**-**d**) stainings in mouse lemurs inoculated with the control brain extract (**a**, **c**) compared with AD-inoculated animals (**b**, **d**). No Aβ and tau deposits were observed in control animals (**a**, **c**) while Aβ (**b**) and tau (**d**) were detected in AD-inoculated animals. Scale bars: 500 µm

Mouse lemurs were euthanized at 21 months post-inoculation (mpi) and their brains were studied by histology. All of AD-inoculated animals (n = 12) developed Aβ deposits (Figs. 1, 2, 3 and 4), whereas none of the Ctrl-inoculated animals displayed any Aβ pathology (n = 6, Fig. 5a–b). These deposits were detected using 4G8 (Fig. 1a, c, g–h) or Aβ42 (Fig. 1b, d) antibodies as well as a Thioflavin S staining (Fig. 1e–f). They occurred in the forms of diffuse (Fig. 1a–b) and dense (Fig. 1c–f) parenchymal Aβ plaques or Aβ angiopathy affecting cortical and hippocampal vessels (Fig. 1g–h), although angiopathy was less prominent than parenchymal deposits. Aβ pathology spread widely throughout the brain and was detected in most cortical regions (Figs. 2, 3, 4a, c–f). Indeed, animals from both AD-inoculated groups showed Aβ deposition in the inoculated posterior cingulate cortex (Figs. 2i, 3a) and/or around the needle tract in the parietal cortex (area 7) (Figs. 2l–n, r, 3b). Adjacent regions including the retrosplenial cortex (Figs. 2q, 3c), anterior cingulate cortex (Figs. 2a, e, 3d) and parietal area 5 (Figs. 2j–k, 3e) also displayed Aβ pathology. Additionally, Aβ plaques were detected in the hippocampus (Figs. 2p, t, 3f) and other regions more distant from the inoculation sites including the superior temporal cortex (areas 22, 21 and 20) (Figs. 2g, o, s, 3g), the parietal cortex involving the primary somatosensory cortex (areas 1–3, Fig. 2c, f) and the frontal cortex, including the primary motor cortex (area 4, Figs. 2b, 3h). Occipital areas also displayed Aβ deposits (area 17, Fig. 2u–v; area 18, Fig. 2w–x). Aβ pathology was not detected in the entorhinal cortex (Fig. 3i) or in deep grey nuclei, brainstem or cerebellum. Quantitative analysis did not reveal any difference between the two AD-inoculated groups (Fig. 3).

### Tau pathology induction and spreading after AD brain inoculation

**Fig. 6:**
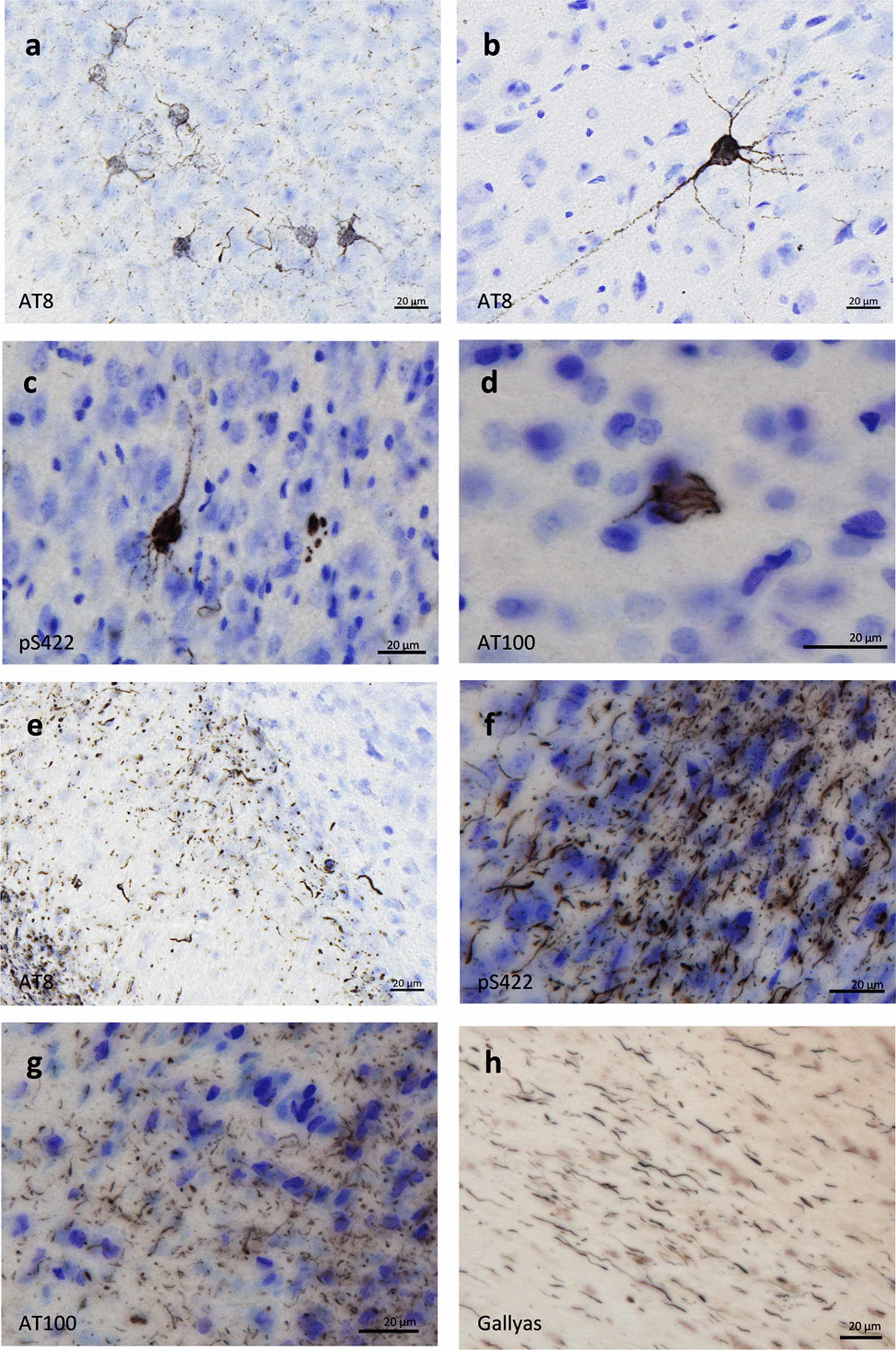
Intraneuronal neurofibrillary tangle and neuropil threads in AD-inoculated animals. AT8-positive neurofibrillary tangle in AD brain inoculed in mouse lemurs were detected after staining with AT8 (**a**-**b**), an antibody detecting phosphorylation of S422 (**c**), and another one targeting phosphorylation of T212 and S214 (AT100) (**d**). Neuropil threads were revealed after staining with AT8 (**c**), anti-pS422 antibody (**f**), AT100 (**g**), and Gallyas staining (**h**). Scale bars: 20 µm

**Fig. 7:**
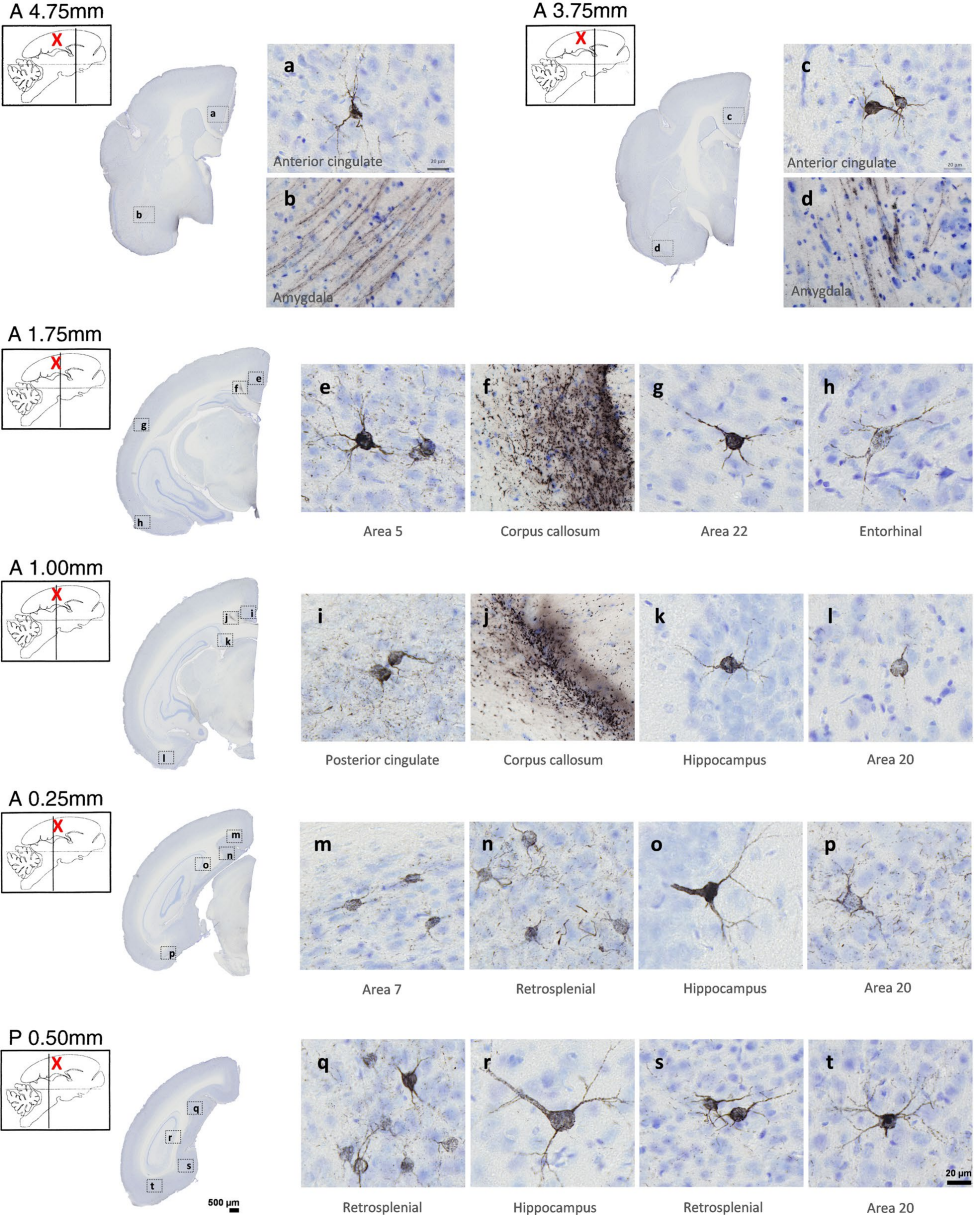
Tau pathology throughout the brains of mouse lemurs inoculated with AD brain extracts*.* Representative images of AT8 immunolabelling showing tau pathology following AD brain extract inoculations in mouse lemurs. NFTs were observed at the level of the inoculation sites, *e.g.* in the posterior cingulate cortex (**i**) and the parietal area 7 (**m**), as well as in juxtaposing regions such as the retrosplenial cortex (**n**, **q**, **s**), anterior cingulate cortex (**a**, **c**), parietal area 5 (**e**). They were also detected within the hippocampus (**k**, **o**, **r**) and temporal area 22 (**g**). Additionally, some regions more distant from the inoculation sites also displayed NFTs, *e.g.* the temporal area 20 (**l**, **p**, **t**) and the entorhinal cortex (**h**). Neuropil threads were mainly observed at the inoculation sites, *e.g.* in the corpus callosum (**f**, **j**) and posterior cingulate cortex (**i**). They were slightly induced around the needle tract (area 7; **m**), in juxtaposing regions such as the retrosplenial cortex (**n**), parietal area 5 (**e**) and in distant regions such as the temporal area 20 (**p**) and the amygdala (**b**, **d**). The red crosses indicate the inoculated region. Scale bars: 500 µm in whole slice images, 20 µm in zoomed images

**Fig. 8:**
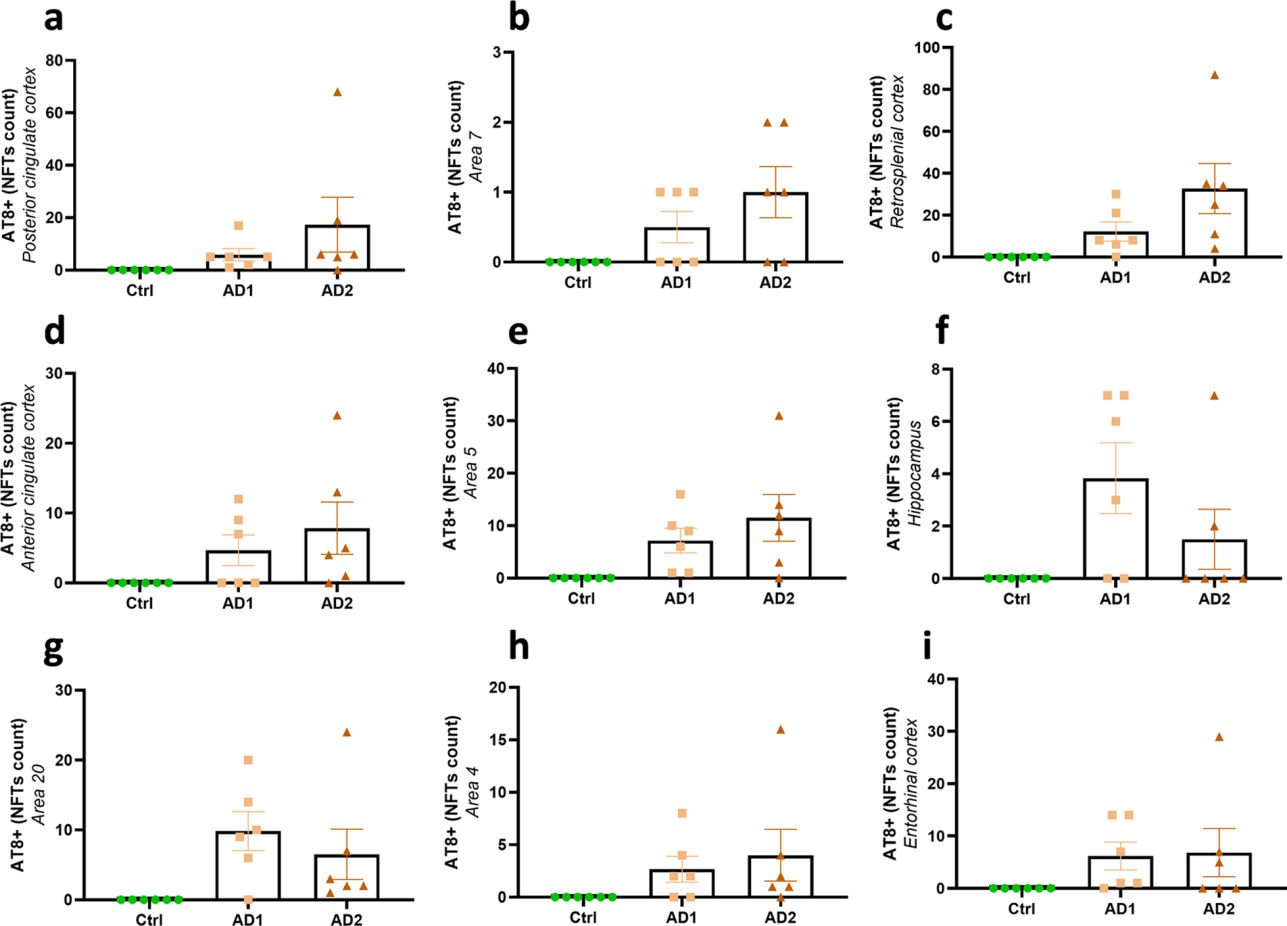
Quantification of NFT pathology following human brain inoculation*.* Quantification of NFT burden (NFT count per region of interest) in AD- and Ctrl-inoculated lemurs. NFTs were detected in all of AD-inoculated animals and in none of Ctrl-inoculated lemurs. In AD-inoculated animals, NFT burden was increased at the level of the inoculation site, *e.g.* in the posterior cingulate cortex (**a**) and the parietal area 7 (**b**), as well as in juxtaposing regions, including the retrosplenial cortex (**c**), anterior cingulate cortex (**d**) and parietal area 5 (**e**). NFTs were also detected in the hippocampus (**f**) and in some regions more distant from the inoculation sites, including the temporal area 20 (**g**), frontal area 4 (**h**) and entorhinal cortex (**i**). No statistical difference was reported between AD1- and AD2-inoculated lemurs (*p* > 0.05; Mann–Whitney’s test). Data are shown as mean ± s.e.m

**Fig. 9:**
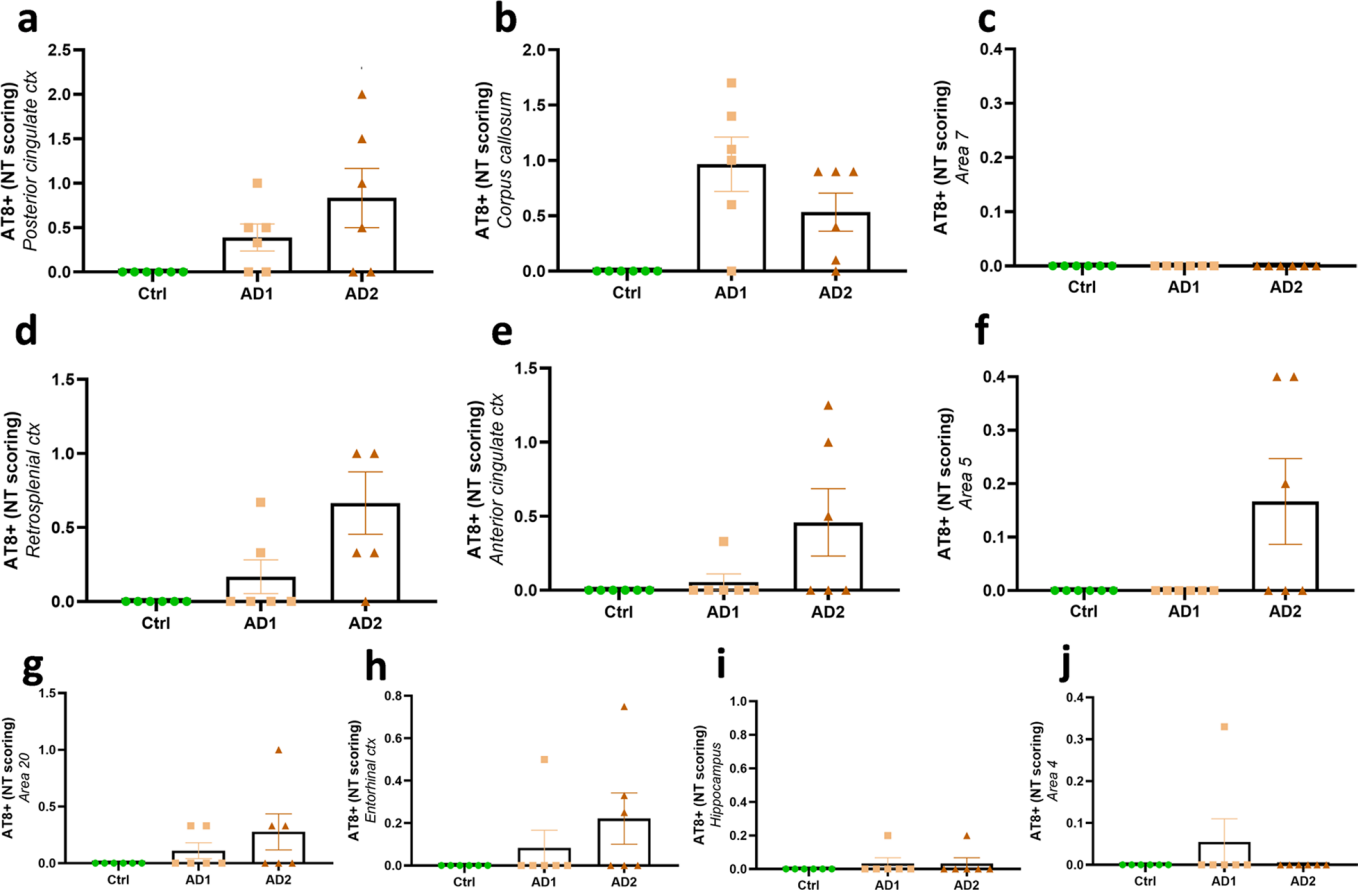
Semi-quantitative scoring of neuropil thread pathology following human brain extracts inoculation*.* Neuropil threads were detected in AD-inoculated animals but not in the Ctrl-inoculated lemurs. AT8-positive neuropil threads were mainly localized at the inoculation sites [posterior cingulate cortex (**a**) and corpus callosum (**b**)], but no lesion was observed around the needle tract (area 7; **c**). They were also induced in juxtaposing regions, such as the retrosplenial cortex (**d**), anterior cingulate cortex (**e**), parietal area 5 (**f**), and in distant regions such as the temporal area 20 (**g**) and entorhinal cortex (**h**). Except for one or two animals, neuropil threads were not detected in the hippocampus (**i**) nor in the frontal cortex (area 4; **j**). No difference was observed between the two AD-inoculated groups (*p* > 0.05; Mann–Whitney’s test). Data are shown as mean ± s.e.m

All AD-inoculated lemurs developed intraneuronal tau accumulations resembling neurofibrillary tangles (NFTs, Fig. 6a–d) and neuropil threads (NTs, Fig. 6e–h), at the inoculation sites and in several other regions (Figs. 4b, g–j, Figs. 7, 8 and 9). Conversely, Ctrl-inoculated animals did not display any tau pathology (Fig. 5c-d). In AD-inoculated animals, tau pathology was detected using AT8 that detects phosphorylation of S202 and T205 [38] (Figs. 6, 7), AT100 that detects phosphorylation at T212 and at S214 that is phosphorylated in AD brains but not in normal brains [38] (Fig. 6d, g), an anti-pS422 antibody (phosphorylation at S422 occurs in AD but not in normal brains [38], Fig. 6c, f), and Gallyas silver staining (Fig. 6h). Neuritic plaques were not detected in any animal. AT8-positive NFTs were localized at the level of the inoculation site (posterior cingulate cortex (Figs. 7i, 8a), parietal area 7 (Figs. 7m, 8b) as well as in juxtaposing regions (retrosplenial cortex (Figs. 7n, q, s, 8c), anterior cingulate cortex (Figs. 7a, c, 8d), parietal area 5 (Figs. 7e, 8e)). They were also detected within the hippocampus (Figs. 7k, o, r, 8f) and temporal area 22 (Fig. 7g). Although temporal regions such as area 21 was never involved, some regions more distant from the inoculation sites displayed NFTs (temporal area 20 (Figs. 7l, p, t, 8g), area 13–16, parietal areas 1–3, frontal area 4 (Fig. 8h) and entorhinal cortex (Figs. 7h, 8i)). Occipital regions (areas 18–17) as well as the most frontal regions did not display tau-positive NFTs, despite the presence of Aβ deposits (Fig. 4). AT8-positive neuropil threads were mainly localized at the inoculation sites (posterior cingulate cortex (Figs. 7i, 8a), corpus callosum (Figs. 7f, j, Fig. 9b), but were barely present around the needle tract (area 7, Figs. 7m, 9c). They were also induced to a lesser degree in juxtaposing regions (retrosplenial cortex (Figs. 7n, 9d), anterior cingulate cortex (Fig. 9e), parietal area 5 (Figs. 7e, 9f)) and in distant regions (temporal area 20 (Figs. 7p, 9g), entorhinal cortex (Fig. 9h) and amydgala (Fig. 7b, d). Except for one or two animals, neuropil threads were not detected in the hippocampus (Fig. 9i) nor in the frontal cortex (area 4, Fig. 9j). As was the case for Aβ, quantitative analysis did not reveal any difference in tau pathologies between the two AD-inoculated groups (Figs. 8 and 9).

### Similar microglial response in AD- and Ctrl-inoculated animals

**Fig. 10:**
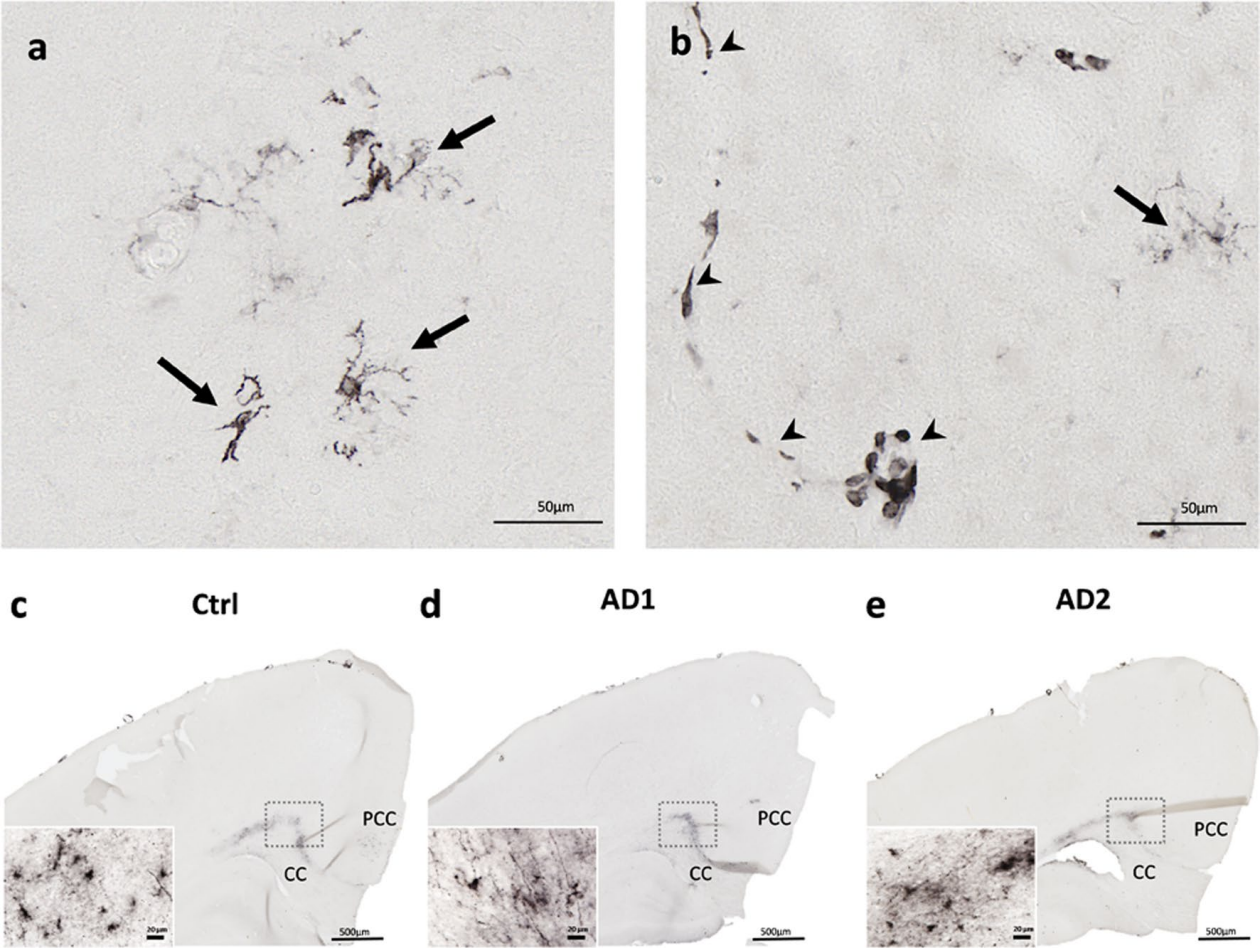
Similar microglial response after the inoculation of Ctrl or AD brain extracts*.* Representative stainings of microgliosis (HLA-DR antibody) in the parenchyma (**a**-**b**, arrows) and in the vasculature (**b**, arrowheads). Staining was observed at the inoculation sites, *i.e.* the posterior cingulate cortex (PCC) and the corpus callosum (CC) (**c**-**e**) but not at distance of these regions. No difference was observed between the groups. Scale bars: 50 µm in **a**-**b**, 500 µm in c-e and 20 µm in inserts

Neuroinflammation was assessed using a histological marker for activated microglia (HLA-DR). Reactive microglia were observed both in the parenchyma (Fig. 10a) and around the vasculature (Fig. 10b) of AD- and Ctrl- inoculated animals. Microglial response mainly involved the inoculation sites (Fig. 10c-e) and was not detected in other regions, even in the presence of Aβ plaques. We did not detect any differences between the groups, suggesting that the inoculation of AD-brain extracts does not induce an exacerbated neuroinflammatory response in comparison with human Ctrl-brain extract.

### Cognitive deficits induced by AD brain inoculation

Learning and long-term memory capabilities were evaluated using discrimination tasks in a jumping stand apparatus [31] before inoculation and at 4, 9, 15 and 21 mpi. The jumping stand apparatus was designed to test the cognition of mouse lemurs using discrimination tasks while taking into account their arboreal lifestyle as well as their sensorial and behavioral skills. At each time-point, a new pair of visual stimuli was introduced to the animals, and learning abilities were evaluated as the lemur had to identify the positive stimulus to reach its nesting box (Additional File 2). Long-term memory was evaluated through the recall of the discrimination task learned during the previous session, 4 to 6 months earlier. At the final timepoint, *i.e.* at 21 mpi, and following a successful discrimination learning session, a reversal learning test was performed to evaluate cognitive flexibility.

**Fig. 11:**
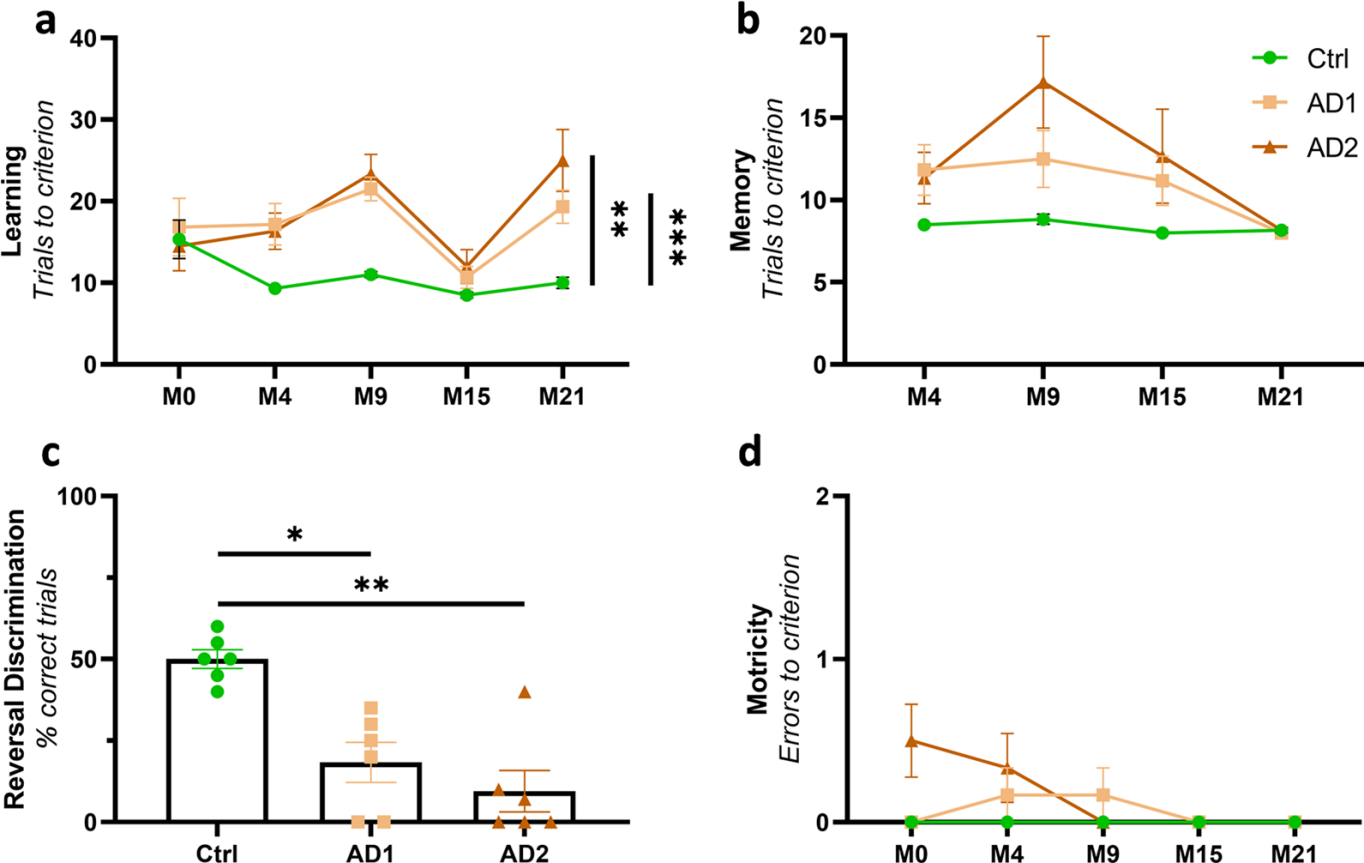
Cognitive impairment induced by AD brain inoculations*.* **a** Longitudinal evaluation revealed learning deficits in AD groups compared to the Ctrl group (Time effect: F_(2.18, 32.73)_ = 8.42, *p* = 0.0009; Group effect: F_(2, 15)_ = 9.52, *p* = 0.002; *p* = 0.0002 and 0.004, respectively for AD1 and AD2 compared to the Ctrl group; two-way ANOVA with the Geisser-Greenhouse correction, Tukey’s multiple comparisons). **b** Memory performance comparison revealed a group effect throughout the follow-up, but no statistical difference between AD1, AD2 and Ctrl animals was detected following post-hoc evaluations (Time effect: F_(2.46, 6.95)_ = 7.40, *p* = 0.001; Group effect: F_(2, 15)_ = 3.83, *p* = 0.045; *p* = 0.066 between AD2 and Ctrl at 9 mpi, p > 0.17 for every other comparisons; two-way ANOVA with the Geisser-Greenhouse correction, Tukey’s multiple comparisons). **c** Reversal learning task performed at 21 mpi revealed deficits in AD groups compared with the Ctrl group (*p* = 0.024 and 0.0045, respectively for AD1 and AD2; Kruskal–Wallis with Dunn’s multiple comparisons). For all of the evaluated cognitive functions, no statistical difference was observed between AD1- and AD2-inoculated lemurs. **d** No motor impairment was observed with age and between the groups all throughout the follow-up (Time effect: F_(2.16, 32.42)_ = 2.35, *p* = 0.11; Group effect: F_(2, 15)_ = 2.57, *p* = 0.11; two-way ANOVA with the Geisser-Greenhouse correction, Tukey’s multiple comparisons). *p < 0.05; **p < 0.01; ***p < 0.001. Data are shown as mean ± s.e.m

AD-inoculated animals showed lower learning abilities in comparison with Ctrl animals (Fig. 11a). Indeed, for the first discrimination task, before the inoculation of the brain extracts (M0), all groups required a similar number of trials to learn the rewarded stimulus (15.3 ± 5.8, 16.8 ± 8.6 and 14.5 ± 7.4 trials for Ctrl, AD1 and AD2 inoculated animals, respectively). Learning performances improved in the Ctrl-inoculated group, reaching the best possible score (at least 8 correct choices out of the first 10 consecutive trials) as early as during the second learning session at 4 mpi (M4), thus demonstrating highly effective acquisition of the learning set. Conversely, learning abilities in the two AD groups declined markedly over time, and performances were significantly worse compared to the Ctrl group throughout the follow-up (Fig. 11a,  *p* = 0.0002 and *p* = 0.004, respectively for AD1 and AD2). Long-term memory performance also differed between the groups throughout the study, but no post-hoc differences were detected between AD-inoculated animals and controls (Fig. 11b). A reversal learning test was performed only at 21 mpi and Ctrl animals performed better than AD1 or AD2-inoculated lemurs (Fig. 11c, *p* = 0.024 and *p* = 0.0045, respectively). For all of the evaluated cognitive functions, no statistical difference was observed between AD1- and AD2-inoculated lemurs (Fig. 11a–c). Altogether, these data suggest that inoculation with AD brain extracts impairs learning abilities as well as cognitive flexibility, while long-term memory is globally preserved. Additionally, motor function was evaluated and showed that, as expected, all animals displayed similar motor skills all throughout the follow-up (Fig. 11d). These data suggest that cognitive deficits observed in AD-inoculated animals in the jumping stand apparatus were not related to motor impairments.

### Progressive cerebral atrophy induced by AD brain inoculation

**Fig. 12:**
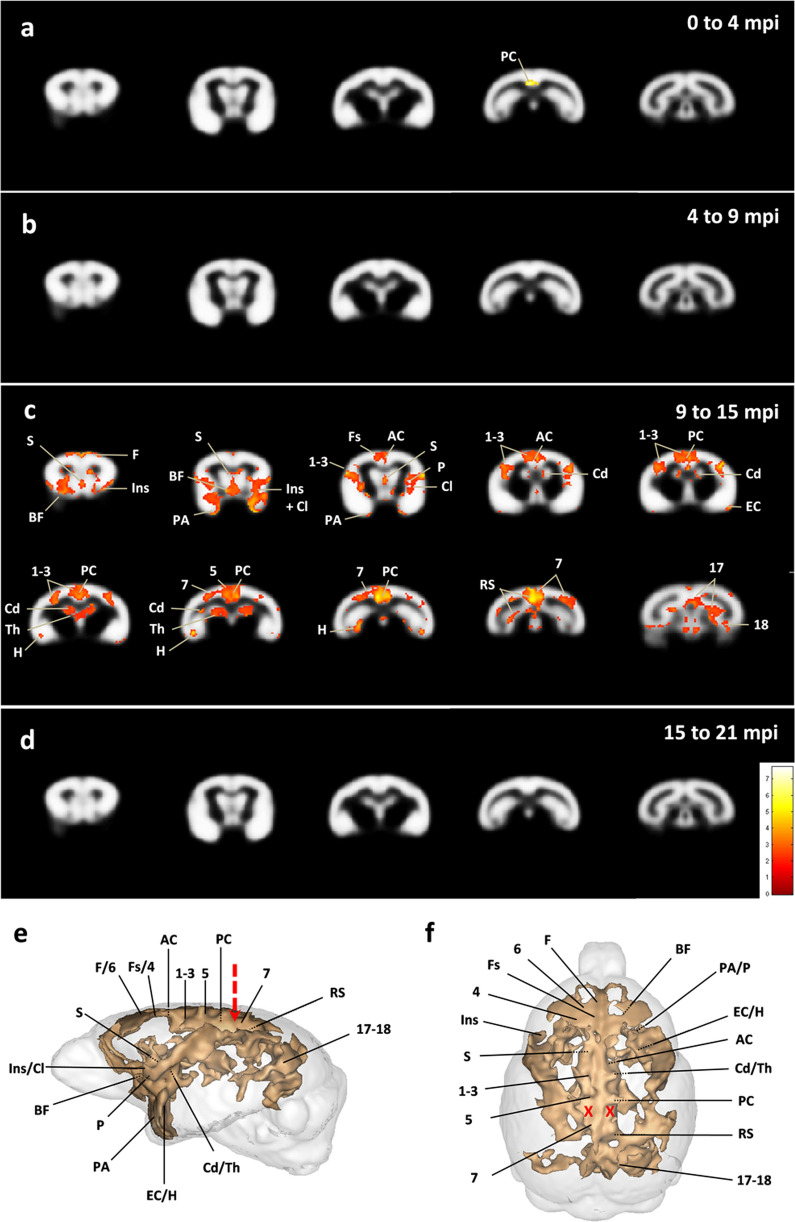
Progressive cerebral atrophy induced by AD brain inoculation*.* Statistical heatmaps of t-values depicting regions in which grey matter volume decreased in AD-inoculated animals compared with Ctrl-inoculated ones (voxel-based morphometry, FDR-corrected *p* < 0.05; voxel threshold extent *k* = 10). To increase statistical power, AD1 and AD2-inoculated animals were grouped within a unique AD group. Longitudinal follow-up revealed a slight atrophy in the inoculated posterior cingulate region at 4 mpi (**a**). No intergroup difference in brain volume was observed between 4 and 9 mpi (**b**). Grey matter loss however extended to several other regions by 15 mpi (**c**), without further progressing afterwards until 21 mpi (**d**). Lateral (**e**) and dorsal (**f**) three-dimensional representations showing clusters of atrophied areas. The red arrow and crosses indicate the injection needle tract. 1–3: cerebral cortex areas 1–3, 4: cerebral cortex area 4, 5: cerebral cortex area 5, 6: cerebral cortex area 6, 7: cerebral cortex area 7, 17–18: cerebral cortex areas 17–18, AC: anterior cingulate cortex, BF: basal forebrain, Cd: caudate nucleus, Cl: claustrum, EC: entorhinal cortex, F: antero-medial frontal cortex, Fs: superior frontal cortex, H: hippocampus, Ins: insular cortex (areas 13–16), P: putamen, PA: peri-amygdalar cortex, PC: posterior cingulate cortex, RS: retrosplenial cortex, S: septum, Th: median thalamus [22]

Brain MRI acquisitions were performed before inoculation and at 4, 9, 15 and 21 mpi. To increase statistical power, AD1 and AD2-inoculated animals were grouped within a unique AD group to be compared with Ctrl-inoculated lemurs. Cerebral atrophy was evaluated using an automated voxel-based morphometry analysis [36]. To control for multiple comparisons, an adjusted p-value was calculated using the voxel-wise false discovery rate (FDR-corrected *p* < 0.05), with extent threshold values of 10 voxels. At 4 mpi, only a slight atrophy was detected in the inoculated posterior cingulate cortex of AD animals, compared to the Ctrl group (Fig. 12a). This atrophy did not progress between 4 and 9 mpi (Fig. 12b), but between 9 and 15 mpi significant bilateral atrophy occurred in several other brain regions (Fig. 12c). It involved the inoculation site, *i.e.* the posterior cingulate cortex, and the parietal cortex (area 7, site with the needle tract) of AD brain-inoculated lemurs (Fig. 12c). Grey matter loss was also observed close to the inoculation sites in the retrosplenial, parietal area 5 and anterior cingulate cortices (Fig. 12c). In addition, cerebral atrophy was reported in several other cortical areas including the prefrontal cortex (antero-medial area), frontal cortex (superior frontal cortex, areas 4 and 6), parietal areas 1–3, temporal cortex (entorhinal and periamygdalar cortices), insular cortex (areas 13–16), and occipital visual cortex (areas 18 and 17) (Fig. 12c). Atrophy was also detected in some subcortical regions such as the hippocampus, the basal forebrain (including the diagonal band of Broca and nucleus accumbens), claustrum, septum, basal ganglia (including the caudate nucleus and putamen) and medial thalamus (Fig. 12c). Atrophy did not further increase from 15 to 21 months post-inoculation (Fig. 12d).

## Discussion

For the first time in a primate, we induced widespread Aβ and tau pathologies along with cognitive impairments and cerebral atrophy following the focal inoculation of AD brain extracts in the cingulate cortex and underlying corpus callosum. These results were replicated using two different batches of AD brain extracts.

Aβ deposits were detected using specific antibodies as well as Thioflavin S staining. They were observed close to the inoculation site and in almost all cortical regions (except for the entorhinal cortex) as well as in the hippocampus, suggesting their efficient spreading within the whole brain. Tau-positive pathology occurred in the forms of neurofibrillary tangles and neuropil threads while neuritic plaques were not detected. They were detected using several antibodies (AT8, AT100, ps422) and Gallyas staining. Tau pathology was evident at the inoculation site and throughout the brain. However some brain regions that were relatively close to the inoculation sites were spared, such as temporal (area 21) and occipital regions (areas 18 and 17). This suggests that the spatial progression of tau did not occur solely via a systematic isotropic diffusion from the injection site to proximal regions. Interestingly, regions such as the entorhinal cortex, that are connected to the cingulate cortex [29] and are distant from the inoculation site were tau-positive but Aβ-negative. This suggests that tau pathology occurred, at least in part, through a transit along neuroanatomical pathways and following different routes as compared to Aβ. Importantly, both Aβ and tau pathologies were detected after a relatively short incubation period of 21 months in young-adult 1.5-year-old primates that are typically devoid of any lesions at this age. We speculate that aggregate deposition might have started even earlier since cerebral atrophy was detected between 9 and 15 mpi.

A chronic neuroinflammatory response, evaluated by HLA-DR staining, was detected at the inoculation sites after human brain extracts inoculation. Such inflammation was not observed in non-inoculated animals (data not shown). Here, inflammation was restricted to the cingulate cortex and underlying corpus callosum, while Aβ and tau pathologies were widespread in the brains of AD-inoculated animals. Also, no difference in microglial activation was observed between the groups of AD- or Ctrl-inoculated animals. This suggests that neuroinflammation cannot explain most of the differences between AD- and Ctrl-inoculated animals. However, one cannot exclude that subtle changes in cell morphology or differences in secreted mediators reflecting different stages of activation could have occurred in AD- and Ctrl-inoculated animals.

Many questions remain following the several neuropathological observational studies in humans showing that Aβ pathology is transmissible [17]. In particular, it is critical to assess whether tau pathology can also be iatrogenically transmitted. Only one recent article suggested that tau could also be detected in patients with iatrogenic Aβ pathology [16]. However this study could not answer if the tauopathy was transmitted or was a consequence of Aβ pathology. Experimental studies in transgenic mouse models have suggested that tau can be transmitted [6], but these models mainly rely on tau protein overexpression. Here, we show definitively that tau pathologies can be transmitted in a primate expressing physiological levels of endogenous tau proteins. Staining for tau pathology was well marked and not necessarily in the direct vicinity of Aβ pathology nor in the same regions. At least three different hypothesis could explain this tau transmission. First, tau seeds from the inoculated brain extracts were responsible for the induction of the tau pathology. This hypothesis is consistent with the fact that brain extracts can induce tau pathologies in mice [7, 11] and that immunodepletion for tau suppresses tau pathology induction [7]. The second hypothesis is that Aβ contained in the human brain extracts (possibly oligomeric forms of Aβ) induced tau pathology in mouse lemur's brain in addition to the induction of Aβ pathology. Thus, tau pathology would not occur through a direct seeding effect of the inoculated human-tau pathology. The lack of co-localization of Aβ plaques (that are known to be also a reservoir for Aβ oligomers) and tau lesions does not support this hypothesis. The third hypothesis is that the inoculated brain extracts contained an undetected compound other than Aβ or tau that induced tau pathology. While we can not rule out this hypothesis, it seems less likely than the first one.

Previous studies have evaluated Aβ and tau pathology induction in primates following the inoculation of AD brain extracts. A 3.5 to 7-year-long study conducted on marmosets intracerebrally inoculated with large volumes of AD brain extracts (300 µl of 10% homogenates distributed within 6 inoculation sites) led to moderate Aβ deposition in most animals but not to tau pathology nor to other AD-like pathological features [2, 23, 34]. In a previous study, our group inoculated AD brain extracts in the hippocampus and overlying cortex of mouse lemurs, leading to sparse Aβ and tau deposits close to the inoculation site [11]. The more severe Aβ and tau pathological inductions observed in the present study, after inoculation of a relatively small volume of brain extracts (25 µl), could in part be explained by variations in brain extract preparations and injection protocols. Here, we sonicated each sample prior to the inoculation as this procedure was shown to enhance the seeding and spreading capacities of brain extracts, supposedly by increasing the level of molecular interfaces for templated misfolding and generating smaller Aβ and tau assemblies that can more readily propagate through the brain than large fibrils [20]. Obviously, Aβ/tau-positive samples that contaminated humans (*e.g.* cadaver-sourced human growth hormones or dura matter) were not sonicated, which could explain much longer incubation times in iatrogenic transmission cases. In addition, in the present study, the injections were performed into highly connected brain areas, such as the corpus callosum and the posterior cingulate cortex. The former is indeed the major commissural tract connecting both hemispheres and the latter is a functional connectivity hub in mouse lemurs [10]. Moreover, the cingulate cortex is involved in the default-mode network, which is disrupted and highly vulnerable to Aβ deposition in AD [5]. Additionally, it is one of the first regions to display atrophy in the course of AD [3].

Whether Aβ and tau iatrogenic transmissions can be associated with clinical impacts in humans remains to be elucidated. Here, along with neuropathological lesions in their brains, AD-inoculated animals displayed cognitive impairments and bilateral brain atrophy. In humans, NFT deposition has been associated with clinical decline [28]. Here, even if we cannot conclude that there is a direct link between Aβ and tau pathologies and cognitive deficits, the occurrence of cognitive alterations suggests that exogenous exposure to misfolded Aβ and tau seeds can translate into clinical manifestations. This is in line with our previous study showing that AD brain extract inoculation in mouse lemurs leads to cognitive impairments despite sparse Aβ and tau deposition [11]. Altogether, this raises concerns about the potential cognitive consequences in patients exposed to contaminated compounds, including vials of human cadaver-derived growth hormones that were shown to contain both Aβ and tau seeds [9, 32].

Cerebral atrophy was prominent close to the inoculation site, *i.e.* the posterior cingulate, where it was first detected at 4 months post-inoculation. Atrophy worsened mainly from 9 to 15 months post-inoculation. This course of atrophy evolution suggests that grey matter loss was not related to an acute toxic effect of the inoculation. Atrophy also gradually spread to several other regions in AD-inoculated animals. Atrophy of the posterior cingulate cortex and connected prefrontal regions [39] and basal ganglia [19] is consistent with deficits in object discrimination learning and cognitive flexibility in AD brain extract-inoculated lemurs, as these structures are thought to play a critical role in instrumental learning and shifting abilities [30, 33, 35].

In conclusion, we provided the first experimental evidence of the transmission of an AD-like phenotype in a non-human primate that includes Aβ and tau pathologies as well as cognitive impairments and cerebral atrophy. This first demonstration of the transmission of tau pathology in a primate calls for further studies in humans to assess the transmission of tau in subjects exposed to contaminated cadaver-derived compounds and surgical material. Our experimental study also outlined the possible consequences of Aβ and tau transmissions in terms of cognitive impairments and cerebral atrophy. Evaluating these aspects of the pathology in patients at risk for Aβ and tau iatrogenic transmission through a systematic monitoring of AD biomarkers is thus urgently needed. Finally, our study also suggests that AD brain-inoculated mouse lemurs are highly relevant models to explore AD pathophysiology and ensure a greater translation of preclinical studies to patients.

## Materials and methods

### Mouse lemurs

Mouse lemurs were reproduced in an approved breeding center (UMR 7179 CNRS/MNHN, France; European Institutions Agreement #962,773) and housed in our laboratory (Commissariat à l'Energie Atomique, Fontenay-aux-Roses center; European Institutions Agreement #B92-032–02). Animals were housed individually in enriched cages containing a wooden nesting box and equipment allowing them to climb and jump freely. The environment was maintained at a constant temperature of 24–26 °C with a relative humidity of 55%. The photoperiodic regime was based on a biannual alternation of long and short days (14 h of light at 250–350 lx/10 h of darkness in the summer period, 10 h of light at 250–350 lx/14 h of darkness in the winter period) in order to artificially reproduce the seasonal rhythm of the animals. Diet consisted of fresh fruits and a preparation based on cottage cheese, eggs, cereals and bananas. Water and food were available ad libitum.

Eighteen males were studied. In order to limit any age-related modifications during the longitudinal follow-up, only young-adults (1.5-year-old) were included. Mouse lemurs were assigned to an experimental group in order to obtain cognitively comparable groups before the inoculations. After human brain inoculation, a 21-month-long follow-up was conducted on the animals. Five other mouse lemurs were evaluated for characterization of tau isoforms by immunoblots. They were sacrificed due to various non-cerebral, non-experimental pathologies (age 9.7 to 10.5 years). All experimental procedures were performed in compliance with the European Union directive on the protection of animals used for scientific purposes (2010/63/EU). They were approved by a local ethics committee (CETEA-CEA DSV IdF) as well as by the French Ministry of Education and Research (authorization A17_083).

### Protein extraction and western blots for mouse lemur brains

Hippocampi and cortices from five non-inoculated mouse lemurs were dissected and snap frozen in 1.5 mL tubes (Eppendorf). Tissus were homogenized in 10 volume of ice-cooled Tris-sucrose buffer (Tris–HCl 10 mM, pH 7.4 10% sucrose added with protease inhibitor (Complete mini EDTA-free, Roche)) and were sonicated (20 pulses of 1 s, amplitude 40%, 60 kHz) on ice. After 1 h at 4 °C, protein concentrations were determined using the BCA Protein Dosage Kit (BioRad, France). Samples were diluted with lithium dodecyl sulphate buffer supplemented with reducing agents (Invitrogen) and then separated on 12% NuPAGE Novex (Invitrogen). Proteins were transferred to nitrocellulose (20 µg for mouse lemurs, 10 µg for control human brains), which were then saturated with 5% non-fat dried milk or 5% bovine serum albumin in TNT (Tris 15 mM pH 8, NaCl 140 mM, 0.05% Tween) and incubated at 4 °C for 24 h with the primary antibodies (TauCter (clone 9F6, LB lab-made), TauNter (12–21, LB lab-made), Tau3R (Millipore ref 05,803), Tau4R (Millipore ref 05,804), TauE2 (Lot1, LB lab-made)). Appropriate HRP-conjugated secondary antibodies (anti- mouse PI-2000 and anti-rabbit PI-1000, Vector Laboratories) were incubated for 45 min at room temperature and signal was visualized using a chemoluminescence kit (ECL, Amersham Bioscience) and a LAS4000 imaging system (Fujifilm).

### Human brain samples

Frozen brain samples (parietal cortex) from clinically different AD patients (four patients with classical slowly evolving forms of AD and four with a rapidly evolving form of AD) and two age-matched control individuals were collected from a brain donation program of the GIE NeuroCEB and the National Reference Center (CNR)-prion brain banks. Consent forms were signed by either the patients themselves or their next of kin in their name, in accordance with French bioethics laws. The classical slowly evolving AD cases (AD1) were characterized by a disease duration of 5 to 8 years. The rapidly evolving AD cases (AD2) were characterized by a disease duration of 6 months to 3 years. No case of hippocampal sclerosis was reported and all brain samples (Ctrl, AD1, AD2) were PrP^Sc^ negative. AD1 and AD2 brain samples were also negative for α-synuclein and TDP-43.

### Neuropathological characterization

All brain tissues were assessed by immunohistochemistry, as previously described in Gary et al. 2019 [11]. Briefly, 4-μm-thick paraffin sections were cut, deparaffinized in xylene, successively rehydrated in ethanol (100, 90, and 70%) and rinsed under running tap water for 10 min before immunohistological staining. They were then incubated in 99% formic acid for 5 min, quenched for endogenous peroxidase with 3% hydrogen peroxide and 20% methanol, and washed in water. Sections were blocked at room temperature for 30 min in 4% bovine serum albumin (BSA) in 0.05 M tris-buffered saline, with 0.05% Tween 20, pH 8 (TBS-Tween, Sigma). They were then incubated overnight at + 4 °C with the 6F3D anti-Aβ antibody (Dako, 1/200), polyclonal anti-tau antibody (Dako, 1/500), monoclonal anti-alpha-synuclein (LB509, Zymed, 1/250), polyclonal anti-TDP43 (Protein Tech Group, 1/1000) routinely used for Aβ, tau, alpha-synuclein and TDP43 detection, respectively. Sections were further incubated with a biotinylated secondary antibody for 25 min at room temperature, and the presence of the secondary antibody was revealed by a streptavidin–horseradish peroxidase conjugate using diaminobenzidine (Dako, Glostrup, Denmark). Sliced were counterstained with Harris hematoxylin.

### Protein extraction for human brains

For tau protein extraction, brain homogenates were sonicated on ice for 5 min, centrifuged for 5 min at 3,000 × g at + 4 °C, diluted in 20 mM Tris/2% SDS and sonicated on ice for 5 min. For Aβ, Iba1 and GFAP protein extractions, brain homogenates were sonicated (6 strokes, cycle 0.5, 30% amplitude) in a lysis buffer at a final concentration of 50 mM Tris–HCl pH 7.4, 150 mM NaCl, 1% Triton-X-100 supplemented with 1X protease inhibitors (cOmplete™ Mini, EDTA-free Protease Inhibitor Cocktail, Roche) and 1/100 diluted phosphatase inhibitors (Phosphatase Inhibitor Cocktail 2, Sigma-Aldrich). Samples were centrifuged at 20,000 × g for 20 min at + 4 °C and the supernatant was collected for further use. Extracted samples were stored at -80 °C after evaluation of total protein concentration by a BCA assay (Pierce™). The obtained concentration was used for the normalization of proteins. Total protein concentration was 5.40, 5.53 and 5.50 µg/µl respectively for the Ctrl, AD1 and AD2 homogenates.

### Western blots for human brain extracts

For tau characterization, samples were diluted to 1 μg/μL, diluted in 2X lithium dodecyl sulfate (LDS, Thermo Fisher Scientific) buffer with reducers and heated at + 100 °C for 10 min. 15 μg of samples were loaded on a 12% Bis-TrisCriterion™ gel (Bio-Rad) and migrated in MOPS buffer for 1 h at 165 V on ice. After protein transfer on nitrocellulose sheets, migration and quality of the transfer were checked with a ponceau S staining. The membrane was saturated for 1 h at room temperature, and was then incubated with the AT100 (pT212-pS214, Life technologies MN1060), 2H9 (pS422, 4BioDx 4BDX-1501), tau-Nter (12–21, LB lab-made) or tau-Cter (clone 9F6, LB lab-made) antibodies overnight at + 4 °C. A peroxidase coupled secondary anti-rabbit or anti-mouse antibody was then applied for 45 min at room temperature. Immunoblotting was revealed by ECL. GAPDH (Sigma 9545) was used as a loading control. For Iba1 and GFAP evaluations, extracted samples were denatured at + 90 °C for 5 min in a buffer containing 1X LDS (NuPAGE® LDS sample buffer, Invitrogen) and DTT 1X (NuPAGE® sample reducing agent, Invitrogen). 10 µg of denatured protein were loaded per well. Samples and molecular weight marker (Bio-Rad Precision Plus Protein™ Dual Color standards) were loaded on 4–20% Criterion™ TGX™ gels (Bio-Rad) and migration was performed in a 1X tris–glycine buffer (Bio-Rad) at 120 V for 1 h. Proteins were then transferred to a nitrocellulose membrane using the Trans-Blot® Turbo™ (Biorad) system. Migration and quality of the transfer were checked with a ponceau S staining. The membrane was then blocked with a TBS/0.1%Tween, 5% milk solution for 1 h at RT, and incubated with the primary antibody Iba1 (Wako 1,919,741, 1/2000), GFAP (Dako Z0334, 1/5000) or actin (Sigma A2066, 1/5000) diluted in saturation buffer overnight at + 4 °C. After washing in TBS/0.1%Tween solution, the membrane was incubated with the appropriate secondary HRP-conjugate antibody diluted to 1/5000 in TBS/0.1%Tween for 1 h at RT. The chemiluminescent signal was revealed using the Clarity western ECL (Bio-Rad) kit and the Chemidoc™ MP (Bio-Rad) imaging system. Protein band intensities were quantified on the ImageJ software and normalized by the actin expression level.

### ELISA quantifications

For Aβ protein quantification, all assay-specific material (pre-coated microtiter plate, buffers, antibodies, standard solutions) was provided in the V-PLEX kit Aβ Peptide Panel 1 (6E10) (MSD®). Human brain homogenates were diluted to 1/5 (Ctrl samples) or 1/10 (AD1 and AD2 samples) in the dilution buffer. As described in the manufacturer's protocol, the microtiter plate was blocked for 1 h at RT with the appropriate buffer. After washing, 25 µl of detection antibody and 25 µl of diluted sample or standard were added in duplicate to the wells and incubated under continuous agitation for 2 h at RT. Wells were washed and 150 µl of reading buffer was added. Plate reading was performed with the MSD Sector Imager 2400 (model 1200) multiplex assay system. Aβ_1-38_, Aβ_1-40_ and Aβ_1-42_ quantifications were performed with the Discovery Workbench 4.0 MSD® software. Tau protein quantifications (total tau and phosphot-tau181) were perfomed according to the manufacturer’s protocol. Briefly, brain homogenates were diluted to 1/100 and 1/200 in the provided dilution buffer. 50 µl of standards or samples, as well as 50 µl of detection antibody solution were added to wells and incubated for 14 h at + 4 °C. After washing, 100 µl of 1X anti-rabbit IgG HRP solution was added for a 30 min incubation period at RT. 100 µl of stabilized chromogen were then added to each well for 30 min at RT, in the dark. The reaction was stopped by adding 100 µl of Stop solution and the plate was read at 450 nm within the hour. Data were analyzed with GraphPad Prism 7 using the 4PL method. All samples were tested in duplicates.

### Human brain extracts preparation

Parietal cortex samples were individually homogenized at 10% weight/volume (w/v) in a sterile 1X Dulbecco’s phosphate buffer solution in CK14 soft tissue homogenizing tubes at 5000 rpm for 20 s (Precellys®, Bertin technologies). Brain extracts were then sonicated on ice for 5 s at 40% amplitude and centrifuged at 3000 g for 5 min at + 4 °C. The supernatant was aliquoted in sterile polypropylene tubes and stored at − 80 °C until use.

Before the stereotaxic injection, 10% Ctrl, AD1 or AD2 individual brain extracts were thawed on ice and combined together according to their group. The three resulting combined samples (Ctrl, AD1 and AD2 brain extracts) were sonicated (70% amplitude, 10 s on/off; Branson SFX 150 cell disruptor sonicator, 3.17 mm microtip probe Emerson, Bron) on ice in a sterile environment.

### Stereotaxic surgery

Stereotaxic surgery was performed to infuse the brain extracts, bilaterally in the posterior cingulate cortex and the underlying corpus callosum. Mouse lemurs have a brain structure that can show significant interindividual variation. The stereotactic injection protocol developed during this study ensures that this variability is taken into account and therefore improves the accuracy of the injections. This protocol is based on a magnetic resonance imaging (MRI) acquisition followed by a surgical procedure. Mouse lemurs were maintained in a stable position on a non-magnetic bed, compatible with the MRI and stereotactic equipment, and for which the positions of the anesthetic mask, muzzle bar and ear bars were adjustable to fit the morphology of each animal. An intra-laboratory validation was performed prior to this study. Animals were fasted the day before the intervention. Water was available ad libitum up to 1 h before anesthesia.

Injection coordinates for the inoculated structures were calculated in reference to a landmark that crossed the middle of the ear bar for the antero-posterior axis, interhemispheric fissure for the left–right axis, and the skull surface for the dorso-ventral axis. We chose this landmark as it could be visualized both in MRI and under the surgeon's binocular magnifying glass. The coordinates of the targeted regions were defined on the MRI and were transposed into stereotaxic coordinates for the surgery.

The MR exams were performed using the following procedure. Mouse lemurs were pre-anesthetized by a subcutaneous injection of glycopyrronium bromide (0.05 ml/kg; Robinul-V®) 30 min before isoflurane anesthesia (induction at 4.5%, maintenance at 1.5%; Vetflurane®). Throughout the procedure, their respiratory rhythm was monitored and body temperature was maintained at 37 ± 0.5 °C using a heated blanket. The MRI system was an 11.7 Tesla Bruker BioSpec (Bruker, Ettlingen, Germany) running ParaVision 6.0.1. Images were acquired using a T2-weighted multi-slice multi-echo sequence with the following parameters: TR = 7100 ms; TE = 24.20 ms; echo spacing = 4.40 ms; echo average = 10; slice thickness = 0.23 mm; number of slices = 128; Axial–AP; Read/Phase/Slice: Y/X/Z; resolution = 0.156 × 0.156 after zero-filling-interpolation (2.5 × 2.5); matrix = 104 × 104; field of view = 40 × 40 mm^2^; bandwidth = 100,000 Hz; acquisition duration: 12min18s.

Just after the MRI, animals were placed in a stereotactic frame (Phymep). Local anesthesia was performed with a subcutaneous injection of lidocaine (5 mg/kg; 0.5% Xylovet, Ceva), after cleaning the incision site with povidone iodine (Vétédine®). Injections were performed bilaterally into the corpus callosum followed by the overlying posterior cingulate cortex, at the previously defined coordinates. Using 1 ml-Hamilton syringes and 34-gauge needles, 6.25 µl of human brain homogenates were administered per injection site, at a 0.5 µl/min rate. At the end of each injection, needles were held in place for five additional minutes before removal. The incised area was cleaned with 10% povidone iodine (Vétédine®) and sutured. Animals then received a subcutaneous injection of a 0.9% sodium chloride solution (1 ml/100 g) for rehydration, after which they were placed in a ventilated heating box (25 °C) and monitored until full recovery from anesthesia. Two weeks after the injection, an MRI was performed to evaluate potential post-operative complications (increased inflammation, cerebral hemorrhage). No imaging abnormalities were detected in any animals.

#### Behavioral tests

Learning, long term memory performance and reversal learning abilities were evaluated using discrimination tests in a jumping stand apparatus [31]. Motor skills were evaluated using a tower test [25]. All tests were conducted using devices adapted to lemurs developed by our team or other mouse lemur specialists.

##### Visual discrimination test in a jumping stand apparatus

The jumping stand apparatus evaluates lemurs' learning and long-term memory performances using discrimination tests [11, 31] (Additional File 2). The device is a vertical cage made of plywood walls, except for the front panel which consists in a one-way mirror allowing observation. During the test, the animal is placed on a elevated central platform and must jump from this starting platform to one of the two landing platforms. If no spontaneous jump is performed within a minute, the central platform can be tilted slightly downwards, creating a slippery slope, to encourage the animal to jump. Each landing platform is associated with a different visual stimulus, characterized by a specific shape, texture and pattern. For each pair of visual stimuli, one is associated with a positive reinforcement (*i.e.* a stable platform giving access to a 2-min rest in a wooden nesting box), whereas the other one is associated with a negative reinforcement (*i.e.* an unstable platform leading to a fall to the bottom of the cage). After a fall, the mouse lemur is left at the bottom of the cage for 20 s before the next trial. As mouse lemurs prefer confined spaces, reaching their nesting box when placed in an open space is a strong motivator for behavioral testing. During a discrimination task, the mouse lemur had to identify the positive stimulus, the location of which was randomly alternated between the two landing platforms over the course of the test and changed at least every three trials. The test ended when the success criterion (*i.e.* at least 8 correct choices out of 10 consecutive attempts) was reached. The score given to an animal during learning and memory tasks was the number of trials required to reach this criterion.

Before the first test, an habituation session consisting of 7 trials was carried out. During the first four trials, only one stable central landing platform was available in front of the nesting box opening. Trial 1 was characterized by the presence of a board directly connecting the departure platform to the landing platform, so that jumping was not required to access the nesting box. This board was removed for trial 2, during which the lemur needed to jump to reach the stable landing platform. During trial 3 and 4, an opaque screen was placed above the landing platform in order to hide the opening of the nesting box. The animal therefore needed to jump onto the stable landing platform and go under the screen to reach the reward. During trial 5 to 7, an unstable platform was introduced, and alternately placed either on the right or on left of the nesting box entrance.

Learning abilities were evaluated as the lemur had to identify the positive stimulus out of the two stimuli to reach its nesting box. Long-term memory performance reflected the ability of mouse lemurs to remember the positive stimulus presented 4 or 6 months earlier. In order to create experimental groups with comparable learning capacities, the learning test was carried out before the inoculation with human brain homogenates (M0). Memory performance was evaluated 4 months later, followed (one day later) by a new learning task. Longitudinal evaluation was performed by a succession of learning and memory tasks at 4, 9, 15 and 21 months post-inoculation. At each timepoint, a new pair of visual stimuli was introduced to the animals for the new learning task. The third cognitive task was a reversal learning task conducted at 21 mpi. After a successful discrimination learning session, the outcomes associated with the two stimuli were reversed. Twenty consecutive trials were performed for each animal and the percentage of correct choices was compared between the groups.

##### Motricity test

The tower test was designed to evaluate motor performances of the lemurs through the achievement of successive high jumps [25]. Similarly to the jumping stand apparatus, it is based on the strong motivation of mouse lemurs to reach their nesting box in order to escape the discomfort felt in open spaces. The device is a 180 × 35 × 35cm tower with three opaque sides and one transparent Plexiglas side. It is crossed horizontally by seven metal rods with a diameter of 5 mm and gradually spaced from one another by 10 cm (at the bottom) to 30 cm (at the top). Jumps with increasing difficulty need to be completed in order to reach the reward. A nesting box can be positioned at three different levels, making it accessible after reaching rod 5 (low position), 6 (intermediate position) or 7 (high position). The test was monitored by a camera placed in front of the tower.

During the test, the lemur was placed on the floor of the tower and had to jump from rod to rod in order to reach the nesting box made accessible after jumping on rod 7. Jumps were either performed spontaneously or after a stimulus in case of immobility (> 3 min). This stimulus could be visual (introduction of a visual stimulus at the bottom of the tower), or mechanical in case of a more prolonged inactivity when on a rod (rotation of the rod on which the lemur was located). When the animal reached the nesting box, it was rewarded with a 5-min rest inside.

When the animal was introduced to the device for the first time, an habituation session consisting of six trials was carried out. Throughout the trials, the nesting box was gradually moved upwards, from the lower position during trial 1 and 2, to the intermediate position during trial 3 and 4, and finally to the upper position during trial 5 and 6.

The motricity test phase consisted of three consecutive trials during which the nesting box was placed at the upper position. The number of falls between the last two rods, spaced by 30 cm, was used as a criterion reflecting motor performances. Motricity tests were performed following the visual discrimination test before humain brain inoculation and at 4, 9, 15 and 21 months post-inoculation.

#### Morphological MRI

Brain volume evolution was evaluated throughout the study, starting before inoculation and then at 4, 9, 15 and 21 months post-inoculation. All images were acquired on a 11.7 Tesla MRI (Bruker Corporation), using the following parameters: TR = 8000 ms; TE = 28.05 ms; echo spacing = 5.10 ms; echo average = 10; slice thickness = 0.23 mm; number of slices = 128, Axial–AP; Read/Phase/Slice: Y/X/Z; resolution = 0.115 × 0.115 after zero-filling interpolation (1.34 × 1.34); matrix = 192 × 192; field of view = 29.44 × 29.44 mm^2^; bandwidth = 100,000 Hz; acquisition duration: 25min36s. Animals were anesthetized and monitored as previously described for stereotaxic injections.

Images were analyzed using voxel-based morphometry by applying SPM8 (Wellcome Trust Institute of Neurology, University College London, UK, www.fil.ion.ucl.ac.uk/spm) with the SPMMouse toolbox (http://spmmouse.org) for animal brain morphometry using a procedure already implemented for mouse lemurs [11, 36]. The brain images were segmented into grey (GM) and white matter (WM) tissue probability maps using locally developed priors, then spatially transformed to the standard space, defined by Sawiak et al., using a GM mouse lemur template [36]. Affine regularization was set for an average-sized template, with a bias non-uniformity FWHM cut-off of 10 mm and a 5 mm basis-function cut off and sampling distance of 0.3 mm. The resulting GM and WM portions were output in rigid template space, and DARTEL [1] was used to create non-linearly registered maps for each subject and common templates for the cohort of animals. The warped GM portions for each subject were adjusted using the Jacobian determinant from the DARTEL registration fields to preserve tissue amounts ("optimized VBM" [13]) and smoothed with a Gaussian kernel of 600 µm to produce maps for analysis.

A general linear model was designed to evaluate relative changes in GM values as a function of time between the control- and Alzheimer's disease-inoculated groups. This type of analysis produces t-statistics and color-coded maps that are the product of a statistical analysis performed at every voxel in the brain. Contiguous groups of voxels that attain statistical significance, called clusters, are displayed on brain images. To control for multiple comparisons, an adjusted p-value was calculated using the voxel-wise false discovery rate (FDR-corrected p < 0.05), with extent threshold values of 10 voxels, meaning that clusters required 10 contiguous voxels to be selected as relevant [12]. Voxels with a modulated GM value below 0.2 were not considered for statistical analysis. The operator was blinded to the group attribution during image processing.

#### Histology

Mouse lemurs were sacrificed at 21 mpi with an intraperitoneal injection of pentobarbital sodium (0.1 ml/100 g; Exagon, Axience). Twenty minutes before sacrifice, a subcutaneous administration of buprenorphine (0.1 mg/100 g; Vétergésic®) was performed for analgesia. Animals were perfused intracardiacally with cold sterile 0.1 M PBS for 4 min, at a rate of 8 ml/min. The brain was post-fixed in 4% paraformaldehyde for 48 h at + 4 °C, transferred in a 15% sucrose solution for 24 h and in a 30% sucrose solution for 48 h at + 4 °C for cryoprotection. Serial coronal sections of 40 µm were performed with a microtome (SM2400, Leica Microsystem) and stored at −20 °C in a storing solution (glycerol 30%, ethylene glycol 30%, distilled water 30%, phosphate buffer 10%). Free-floating sections were rinsed in a 0.1 M PBS solution (10% Sigma-Aldrich® phosphate buffer, 0.9% Sigma-Aldrich® NaCl, distilled water) before use.

##### Immunohistology, Thioflavin S and Gallyas silver staining

Mouse lemur brain sections were stained for amyloid-β (4G8 and Aβ42 antibodies, Thioflavin S), tau (AT8, AT100, pS422 antibodies and Gallyas silver staining) and microglia (HLA-DR antibody). A pretreatment with 70% formic acid (VWR®) for 20 min at room temperature (RT) was performed for the 4G8 labelling. For the HLA-DR and AT100 stainings, the pretreatment consisted in a 30 min-long incubation in EDTA 1X citrate (Diagnostic BioSystems®) at 95 °C followed by rinsings in 0.5% Triton X-100/0.05 M Tris–HCl Buffered Saline solution (TBS) or a PBS solution respectively, for 2 × 10 min. For the 4G8, Aβ42, AT8, AT100, pS422 and HLA-DR stainings, tissues were then incubated in hydrogen peroxide H_2_O_2_ 30% (Sigma-Aldrich®) diluted 1/100 for 20 min to inhibit endogenous peroxidases. Blocking of non-specific antigenic sites was achieved over 1 h using a 0.2% Triton X-100/0.1 M PBS (Sigma-Aldrich®) (PBST) or 0.5% Triton X-100/0.05 M TBS (TBST) solution containing 3% bovine serum albumin or 4.5% normal goat serum, depending on the staining. Sections were then incubated at + 4 °C with the 4G8 (Biolegend 800,706, 1/350), Aβ42 (Invitrogen 44,344, 1/500) or pS422 (Abcam 79,415, 1/1000) antibody diluted in a 3%BSA/PBST solution for 48 h or for 96 h with the AT8 antibody (Thermo MN1020B, 1/500), in a 3%BSA/TBST solution with the HLA-DR antibody (Dako M0746) for 48 h or in a 5%NGS/TBST solution with the AT100 antibody (Thermo MN1060, 1/250) for 96 h. After rinsing, an incubation with the appropriate biotinylated secondary antibody diluted to 1/1000 in PBST or TBST was performed for 1 h at RT, followed by a 1 h incubation at RT with a 1:250 dilution of an avidin–biotin complex solution (ABC Vectastain kit, Vector Laboratories®). Revelation was performed using the DAB Peroxidase Substrate Kit (DAB SK4100 kit, Vector Laboratories®). Sections were mounted on Superfrost Plus slides (Thermo-Scientific®). For the 4G8, Aβ42, AT8, AT100 and pS422 labellings, a cresyl violet counterstain was performed. All sections were then dehydrated in successive baths of ethanol at 50°, 70°, 96° and 100° and in xylene. Slides were mounted with the Eukitt® mounting medium (Chem-Lab®).

For the Thioflavin S staining, free-floating sections were first mounted on Superfrost Plus (Thermo-Scientific®) slides and dried overnight. Sections were then incubated with a Thioflavin S (Sigma T1892) solution for 20 min at RT. They were then incubated in ethanol 100° and mounted with the FluorSave medium.

Free-floating sections were mounted on Superfrost Plus (Thermo-Scientific®) slides and dried overnight prior to Gallyas staining. All steps of the Gallyas staining were performed between 20 and 25 °C. Section were permeabilized by successive incubations in toluene (2 × 5 min) followed by ethanol at 100°, 90° and 70° (2 min per solution). Slides were then incubated in a 0.25% potassium permanganate solution for 15 min, in 2% oxalic acid for 2 min then in a lanthanum nitrate solution (0.04 g/l lanthanum nitrate, 0.2 g/l sodium acetate, 10% H2O2 30%) for 1 h to reduce non-specific background. Several rinses with distilled water were performed followed by an incubation in an alkaline silver iodide solution (3.5% AgNO3 1%, 40 g/l NaOH, 100 g/l KI) for 2 min. The reaction was neutralized with 0.5% glacial acetic acid baths (3 × 1 min) and sections were incubated for 20 min in a developing solution (2 g/l NH4NO3, 2 g/l AgNO3, 10 g/l tungstosilicilic acid, 0.76% formaldehyde 37%, 50 g/l anhydrous Na2CO3). Several rinses with 0.5% acetic acid (3 × 1 min) followed by an incubation in 1% gold chloride solution for 5 min were then carried out. Sections were rinsed with distilled water and the staining was fixed with a 1% sodium thiosulfate solution. All sections were then rinsed with distilled water and dehydrated for 1 to 5 min in successive baths of ethanol at 50°, 70°, 96° and 100° and in xylene. Slides were mounted with Eukitt® mounting medium (Chem-Lab®).

#### Image analysis

Z-stack images were acquired at 20 × (z-stacks with 16 planes, 1 µm steps with extended depth of focus) using an Axio Scan.Z1 (Zeiss®). Each slice was extracted individually in the.czi format using the Zen 2.0 (Zeiss®) software. Image processing and analysis were performed with the ImageJ software (https://imagej.nih.gov/ij/). Images were imported with a 50% reduction in resolution (0.44 µm/pixel), converted to the RGB format and compressed in.tif format.

For the 4G8 immunostaining, the blue component of each image was extracted along with the background in order to remove the cresyl violet counter-staining from the analysis. Segmentation was performed with a manual threshold set at 202/255. Clusters of pixels with a radius below 15 µm were removed. Aβ load was evaluated as a percentage of 4G8-positive surface area in each ROI, except for the hippocampus. In the hippocampus, the number of plaques was manually counted as Aβ deposition was mainly concentrated in some areas and the evaluation of an overall staining was not appropriate. For the AT8 staining, AD-like neurofibrillary tangles quantification was performed by manually counting the lesions and neuropil threads were evaluated by using a semi-quantitative scoring system based on the intensity and extent of the lesions (0 = absent, 1 = slight to moderate, 2 = moderate to severe). For the HLA-DR staining, a semi-quantitative analysis was performed by assigning a severity score based on the intensity and extent of the staining at the inoculation sites (0 = absent, 1 = slight, 2 = moderate, 3 = severe). All quantifications were performed on adjacent slices between A4.50 mm and P1.50 mm from bregma. 4G8 and AT8 were analyzed on 10 slices, HLA-DR on 3 slices adjacent to the inoculation site. ROIs were manually segmented using a mouse lemur atlas previously presented by our group [27], which was based on the reference atlases by Le Gros Clark [22] and Bons [4].

The three-dimensional rendering of Aβ and tau pathologies in mouse lemur brains was based on regions of interests defined in a digital atlas of mouse lemurs [27]. Aβ or tau lesions were reported within regions of interests using a three-level semi-quantitative scale (no lesions/intermediate/high lesion load). The ITK-SNAP software (http://www.itksnap.org) was used to create the surface/2D renderings of lesion loads.

#### Statistical analysis

Statistical analysis was performed using the GraphPad Prism software 9. For human brain characterization, data are shown as mean ± standard deviation of the replicates. Mouse lemur histological data are shown on scattered dot plots with mean ± standard error of the mean (s.e.m.) and Mann–Whitney’s test was carried out to compare results between the AD1 and AD2 groups. Behavioral studies data are shown as mean ± standard error of the mean (s.e.m) to represent performance evolution over time. Cognitive and motor performances which included repeated measures were evaluated using a two-way repeated measures ANOVA with the Geisser-Greenhouse correction and Tukey’s multiple comparisons. The significance level was set at *p* < 0.05.

## Supplementary Information

**Additional file 1**. Supplementary table and figures.

**Additional file 2**. Movie showing three consecutive trials in a jumping stand apparatus.
