# Supplementary material for: Transmission of amyloid-beta and tau pathologies is associated with cognitive impairments in a primate
Source: Acta Neuropathol Commun. 2021 Oct 12;9:165. doi: 10.1186/s40478-021-01266-8 (PMC8507137; doi:10.1186/s40478-021-01266-8)
Supplement: Supplementary file 1 — Additional file 1. Supplementary table and figures. [file 40478_2021_1266_MOESM1_ESM.pdf]

Transmission of amyloid-beta and tau pathologies is associated with cognitive impairments in a primate

Suzanne Lam, Fanny Petit, Anne-Sophie Hérard, Susana Boluda, Sabiha Eddarkaoui,

Martine Guillermier, The Brain Bank Neuro-CEB Neuropathology Network, Luc Buée,

Charles Duyckaerts, Stéphane Haïk, Jean-Luc Picq, Marc Dhenain

Supplementary material

Additional file 1

SUPPLEMENTARY TABLES

Supplementary Table 1

Patient				Neuropathology							
ID	Age	Gender	Disease progression (months)	Post mortem delay (hours)	Braak and Braak stage	Thal phase	CAA	α-synuclein	TDP43	Hippocampal sclerosis	PrPSc
1-Ctrl	66	M	-	26	II	0	0	0	0	0	0
2-Ctrl	76	M	-	NA	II	0	0	0	0	0	0
1-AD1	79	F	78	49	V	5	Type2	0	0	0	0
2-AD1	87	F	72	29	V-VI	4	Type1	0	0	0	0
3-AD1	89	F	96	31	V	5	Type1	0	0	0	0
4-AD1	71	M	66	54	VI	4	Type2	Amygdala	0	0	0
1-AD2	84	F	6	79	V	4	Type2	0	0	0	0
2-AD2	81	F	36	NA	V	5	Type1	0	0	0	0
3-AD2	81	F	36	26	VI	4	0	0	0	0	0
4-AD2	86	F	36	21	V	4	Type2	0	0	0	0

Supplementary Table 1: Patient characteristics. Age-matched slowly (AD1) and rapidly (AD2) evolving AD patients were selected based on disease duration (over or under 36 months) and neuropathological evaluation, including similar Braak and Thal stages. Brains were negative for α-synuclein, TDP43, hippocampal sclerosis and pathological prion PrPSc. Two non-AD control individuals were also included in this study. NA: not available.

SUPPLEMENTARY FIGURES

Supplementary Figure 1

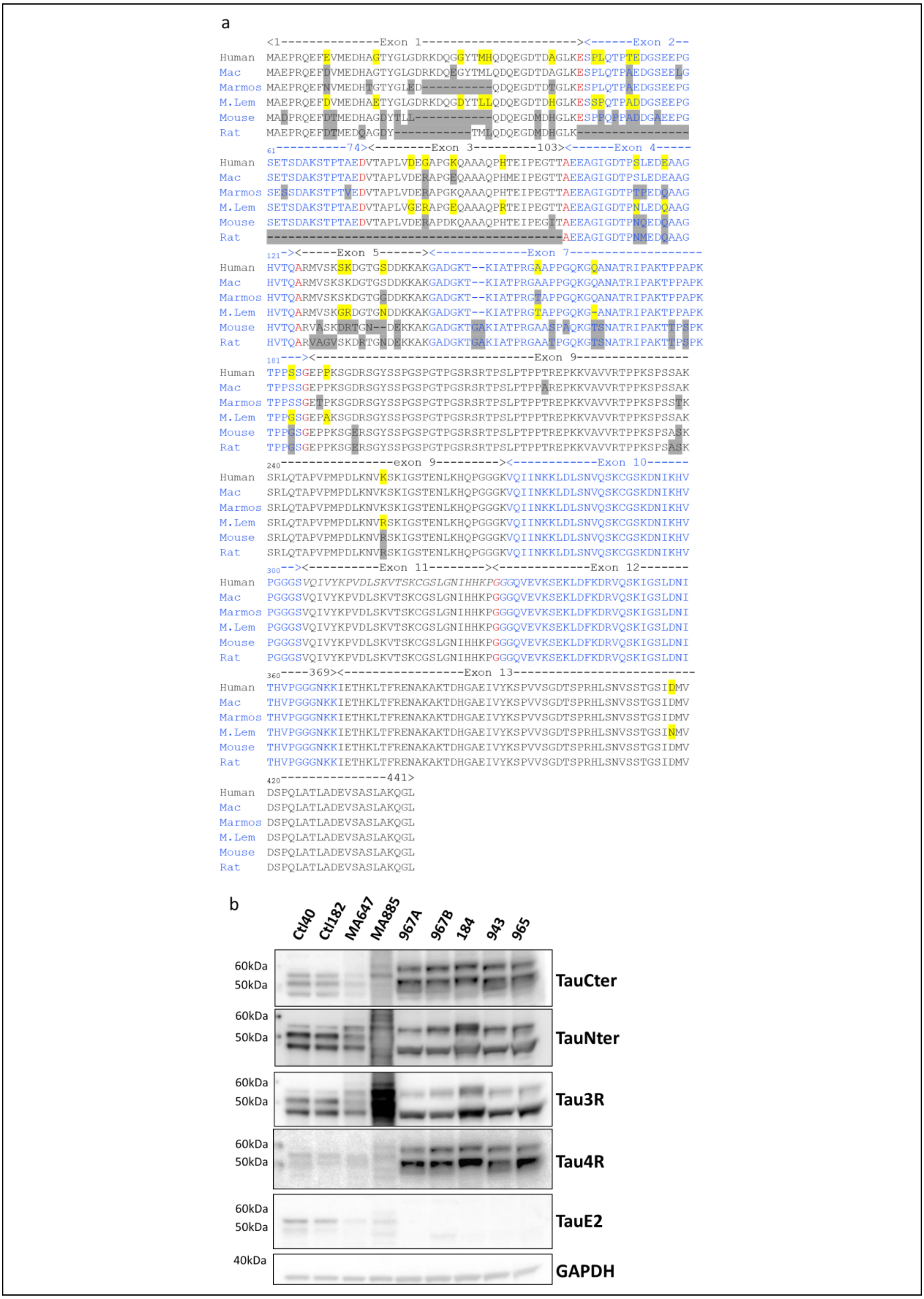

Supplementary Figure 1: Protein sequence issued from MAPT gene in mouse lemurs and tau protein isoforms. (a) Comparison of proteins sequences for MAPT genes (issued from http://www.ensembl.org/) in humans (MAPT-204 ENST00000351559.10), macaques (Mac: MAPT-204 ENSMMUT00000005855.4), marmosets (Marmos: MAPT-202 ENSCJAT00000039192.4), mouse lemurs (M. Lem: MAPT-201 ENSMICT00000067450.1), mice (Mapt-201 ENSMUST00000100347.11), and rats (Mapt-201 ENSRNOT00000006947.8). Different exons are colored black and blue. Residue overlaps splice sites are labelled in red. Differences between humans and mouse lemurs are displayed in yellow. Differences between humans and other species are displayed in gray. (b) Tau isoform expression was examined by immunoblots using antibodies specific for tauCter, tauNter, tau3R, tau4R, and tauE2. TauCter, tauNter, tau3R, and tau4R were detected in the five (non-inoculated) mouse lemurs evaluated in this study (967A, 967B, 184, 943, 965). Isoforms corresponding to exon 2 were not detected.

Supplementary Figure 2

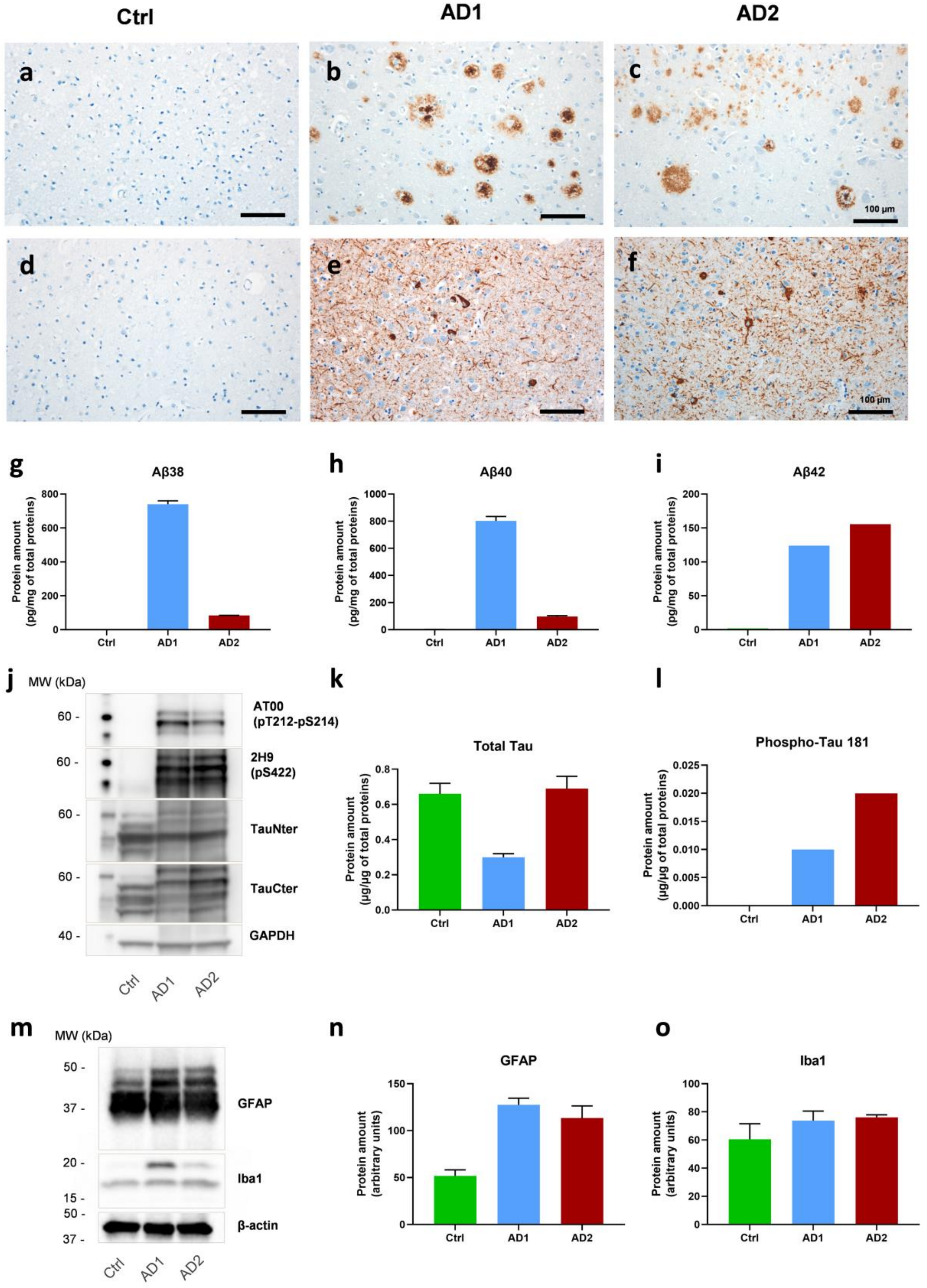

Supplementary Figure 2: Characteristics of human brain samples and brain extracts inoculated to animals. Representative images of Ctrl, AD1 and AD2 brain samples stained

for Aβ (a-c) and tau (d-f) pathologies. Scale bars = 100 µm. Three brain extracts were prepared from 2 control individuals, 4 cases with a slowly evolving form of AD and 4 cases with a rapidly evolving form of AD (Ctrl, AD1 and AD2 brain extracts, respectively). All quantifications of the brain extracts (Ctrl, AD1 or AD2) were performed in duplicate and data are shown as mean ± standard deviation of the replicates. (g-i) Quantifications of total $A\beta_{38}$, $A\beta_{40}$ and $A\beta_{42}$ of the brain extracts (MSD technology). Both AD brain extracts had more Aβ proteins compared to the Ctrl one. The AD1 extract showed more $A\beta_{38}$ and $A\beta_{40}$ than the AD2 one. (j-l) Tau profile evaluation by western blot revealed a pathological hyperphosphorylated tau triplet at 60, 64 and 69 kDa observed in AD and a typical shift in the molecular weight of the Alzheimer Tau-Cter triplet in AD1 and AD2 brain extracts (j). Total tau (k) and pathological phospho-tau 181 levels (l) were assessed using ELISA quantification. Neuroinflammatory profile evaluation by western blots revealed higher astrocytic presence (GFAP-positive) in AD1 and AD2 brain extracts compared to the Ctrl extract (m-n). Microglial (Iba1-positive) levels were similar in the Ctrl, AD1 and AD2 groups (m, o).

Supplementary Figure 3

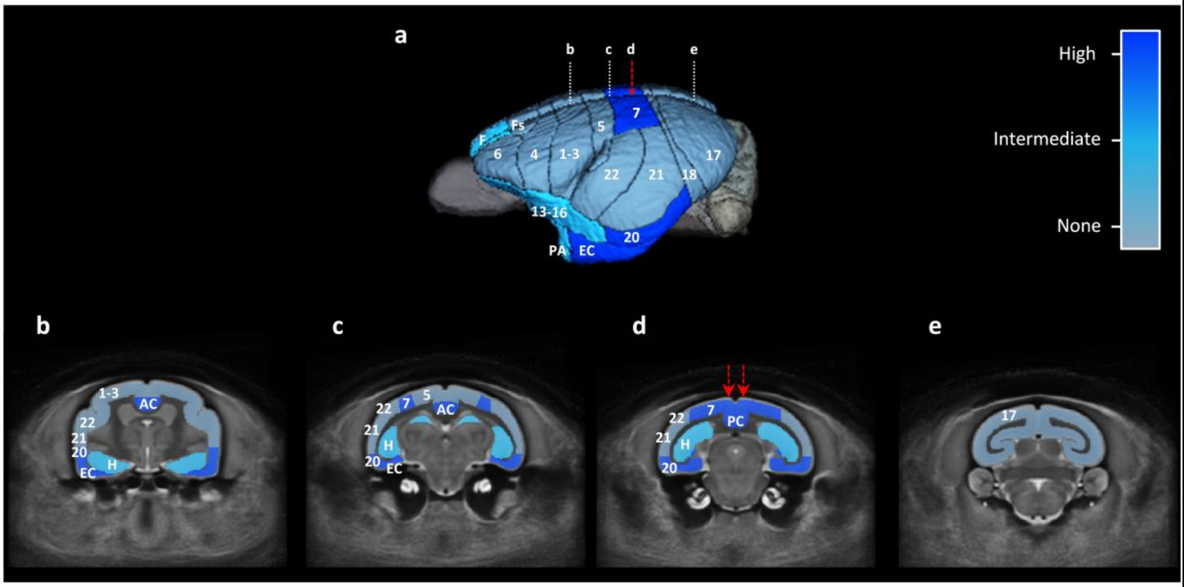

Supplementary Figure 3: Connectivity of the cingulate cortex in mouse lemurs. Three-dimensional rendering of regions connected to the cingulate cortex. Regions are classified according to three levels of connectivity (not connected/intermediate/high connectivity). Connectivity was determined based on literature in primates (Vogt, 1987; Parvizi, 2006). Three-dimensional rendering relied on a digital atlas of mouse lemurs (Nadkarni, 2019) and on the use of the ITK-SNAP software (http://www.itksnap.org). The red arrows indicate the needle tracts. Numbers represent Brodmann areas as reported in the mouse lemur brain by (Le Gros Clark, 1931). AC: anterior cingulate cortex, EC: entorhinal cortex, F: antero-medial frontal cortex, Fs: superior frontal cortex, H: hippocampus, PA: peri-amygdalar cortex, PC: posterior cingulate cortex.

References

- Vogt BA, Pandya DN (1987) Cingulate cortex of the Rhesus-monkey .2. Cortical afferents. J Comp Neurol 262: 271-289. https://doi.org/DOI 10.1002/cne.902620208

- Parvizi J, Van Hoesen GW, Buckwalter J, Damasio A (2006) Neural connections of the posteromedial cortex in the macaque. Proc Natl Acad Sci USA 103: 1563-1568. https://doi.org/10.1073/pnas.0507729103

- Nadkarni NA, Bougacha S, Garin C, Dhenain M, Picq JL (2019) A 3D population-based brain atlas of the mouse lemur primate with examples of applications in aging studies and comparative anatomy. Neuroimage 185: 85-95. https://doi.org/10.1016/j.neuroimage.2018.10.010

- Le Gros Clark WE (1931) The brain of *Microcebus murinus*. Proc Zool Soc London 101: 463-486.

Additional file 2 (movie to download)

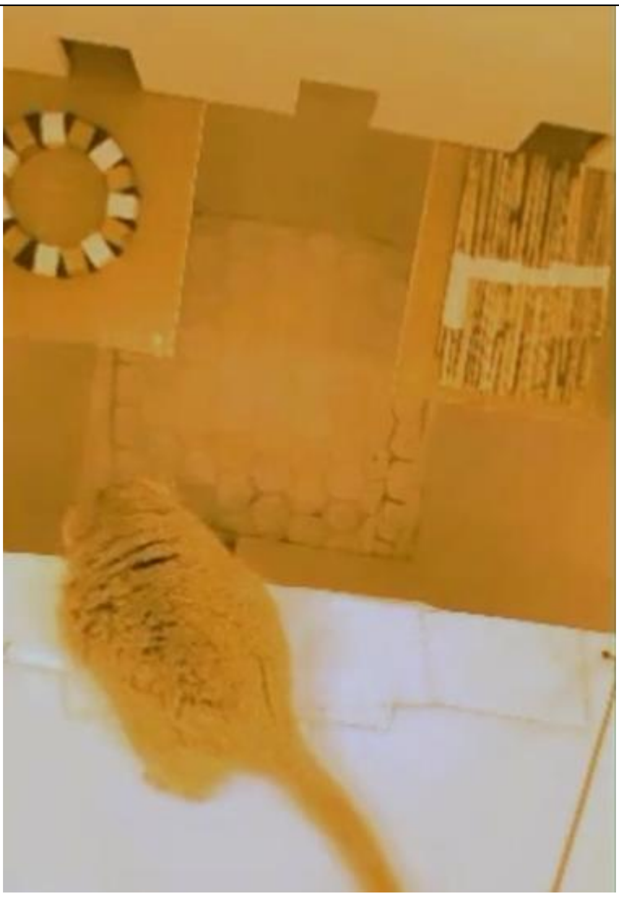

Additional File 2: Movie showing three consecutive trials in a jumping stand apparatus. The jumping stand apparatus evaluates learning and long-term memory performances of lemurs using discrimination tests. The device is a vertical cage made of plywood walls, except for the front panel which consists in a one-way mirror allowing observation. During the test, the animal is placed on a elevated central platform (10 first seconds of the movie) and must jump from this starting platform to one of the two landing platforms. Each landing platform is associated with a different visual stimulus, characterized by a specific shape, texture and pattern (circular object versus rectangular straw-mat in this example). For each pair of visual stimuli, one is associated with a positive reinforcement (*i.e.* a stable landing platform giving access to a 2-min rest in a wooden nesting box), whereas the other one is associated with a negative reinforcement (*i.e.* an unstable landing platform leading to a fall to the bottom of the cage). The mouse lemurs has to learn the rule by trial and error. In this movie, during the first trial, the mouse lemur makes the wrong choice by jumping towards

the straw-mat and falls. During the second trial, it changes its strategy and jump towards the circular object. It keep the same strategy during the third trial. In this movie, one can see how the lemur observes the different objects before its jump.
